# TNF-driven intracellular complement factor H–NF-κB feed-forward loop amplifies inflammatory mediator production in rheumatoid arthritis synovial fibroblasts

**DOI:** 10.1186/s41232-026-00437-1

**Published:** 2026-08-04

**Authors:** Hiroki Kobayashi, Sho Mokuda, Michinori Ishitoku, Hirofumi Watanabe, Tomohiro Sugimoto, Yusuke Yoshida, Junya Masumoto, Shintaro Hirata

**Affiliations:** 1 Department of Clinical Immunology and Rheumatology, Hiroshima University Hospital, Hiroshima, 734-8551 Japan; 2 Division of Laboratory Medicine, Hiroshima University Hospital, Hiroshima, 734-8551 Japan; 3 Department of Pathology, Ehime University Proteo-Science Center and Graduate School of Medicine, Shitsukawa, Toon, Ehime 791-0295 Japan

**Keywords:** TNF signaling, Rheumatoid arthritis, Fibroblast-like synoviocytes, Complement factor H, Intracellular inflammatory amplification

## Abstract

**Background:**

Rheumatoid arthritis (RA) involves multiple pro-inflammatory cytokines; fibroblast-like synoviocytes (FLS) are central effector cells that integrate these signals to produce pathogenic mediators. Tumor necrosis factor (TNF) is a major upstream driver of coordinated inflammatory mediator induction in FLS; however, the underlying mechanisms remain incompletely defined, particularly intracellular feed-forward amplification sustaining and enhancing TNF-driven transcriptional outputs. Complement factor H (FH; gene *CFH*), a Sushi domain-only complement regulator, is elevated in RA serum. FH is regarded as an extracellular inhibitor of complement activation; however, its potential intracellular expression and functions in FLS have not been explored. We aimed to investigate intracellular FH expression and function using an in vitro transcribed messenger RNA (IVT mRNA)-based approach.

**Methods:**

FH expression in sera, synovial tissues, and fibroblast-like synoviocytes (FLS) were assessed by enzyme-linked immunosorbent assay (ELISA), immunohistochemistry, RT-qPCR, and immunoblotting. To examine intracellular FH function, FLS were transfected with *CFH*-encoding IVT mRNA to express FH intracellularly, followed by next-generation sequencing (RNA-seq), RT-qPCR, ELISA, phosphorylation assays, and pathway inhibition. Interactions between TNF-driven signaling and intracellular FH were evaluated using TNF- and/or FH-neutralizing antibodies, with or without *CFH*-targeting small interfering RNA (siRNA).

**Results:**

FH was highly expressed in RA synovium and predominantly localized intracellularly in FLS. To assess intracellular FH function, FLS were transfected with *CFH*-encoding IVT mRNA, which induced the coordinated upregulation of RA-relevant mediators, including granulocyte–macrophage colony-stimulating factor (GM-CSF), interleukin (IL)-8, IL-6, and multiple chemokines. Mechanistically, intracellular FH activated nuclear factor-κB (NF-κB) signaling, promoting receptor-interacting serine/threonine-protein kinase 2 (RIPK2) phosphorylation and p65 nuclear translocation, and the competitive nucleotide-binding oligomerization domain 2 (NOD2) antagonist suppressed FH-driven gene induction. TNF and intracellular FH additively enhanced pathogenic mediator production. *CFH*-targeting siRNA attenuated TNF-induced cytokine and chemokine expression; blockade of extracellular FH had no detectable effect. Combined TNF neutralization and *CFH* knockdown produced more effective suppression of cytokine induction than either intervention alone.

**Conclusions:**

Intracellular FH is a previously unrecognized amplifier of TNF-driven inflammation in RA-FLS via a NOD2/RIPK2/NF-κB axis, establishing a feed-forward loop boosting pathogenic mediator production. Dual targeting of TNF and intracellular FH may represent a therapeutic strategy to suppress synovial inflammation more effectively.

**Supplementary Information:**

The online version contains supplementary material available at https://doi.org/10.1186/s41232-026-00437-1.

## Background

Rheumatoid arthritis (RA) is a chronic inflammatory disease of the synovium that affects multiple joints and leads to progressive bone and cartilage destruction; therapeutic benefits can be achieved by targeting pro-inflammatory cytokines and immune effector cells [1]. Evidence from preclinical, early, and chronic phases of RA suggests that the inflammatory environment changes across disease stages, with reported alterations in the expression and/or circulating levels of several cytokines, including tumor necrosis factor (TNF), interleukin (IL)−6, granulocyte–macrophage colony-stimulating factor (GM-CSF), IL-8, and IL-17 [2, 3]. In parallel, anti-cytokine therapies have been developed for RA, with biologics targeting TNF and IL-6 now established as antirheumatic agents [4, 5]. Therapeutic strategies targeting GM-CSF or IL-17 have been evaluated in clinical trials; however, their clinical positioning has not been clearly established and they have not been incorporated into standard-of-care regimens [6, 7]. Together, these observations indicate the need to elucidate how cytokine networks are regulated and connected to drive the coordinated induction of inflammatory mediators in RA synovium.

Recently, analyses of fibroblast-like synoviocytes (FLS), a major cellular component of synovial tissue, have revealed their crucial role in RA pathogenesis [8, 9]. Cytokines such as TNF and IL-17 activate FLS and induce the production of soluble mediators, including IL-6, GM-CSF, IL-8, chemokines, matrix metalloproteinases (MMPs), and receptor activator for nuclear factor-κB ligand (RANKL), in a subset-dependent manner [10]. Numerous studies have described the regulatory interactions among these cytokines, including the ability of TNF to induce IL-6 expression in FLS [11, 12]. In addition, TNF and IL-17A have been shown to synergistically enhance downstream gene expression in FLS [13–15]. Collectively, these findings position TNF as a major upstream driver of coordinated inflammatory mediator induction in FLS; however, the underlying mechanisms remain poorly defined, particularly intracellular feed-forward amplification that sustain and enhance TNF-driven transcriptional outputs.

**Fig. 1:**
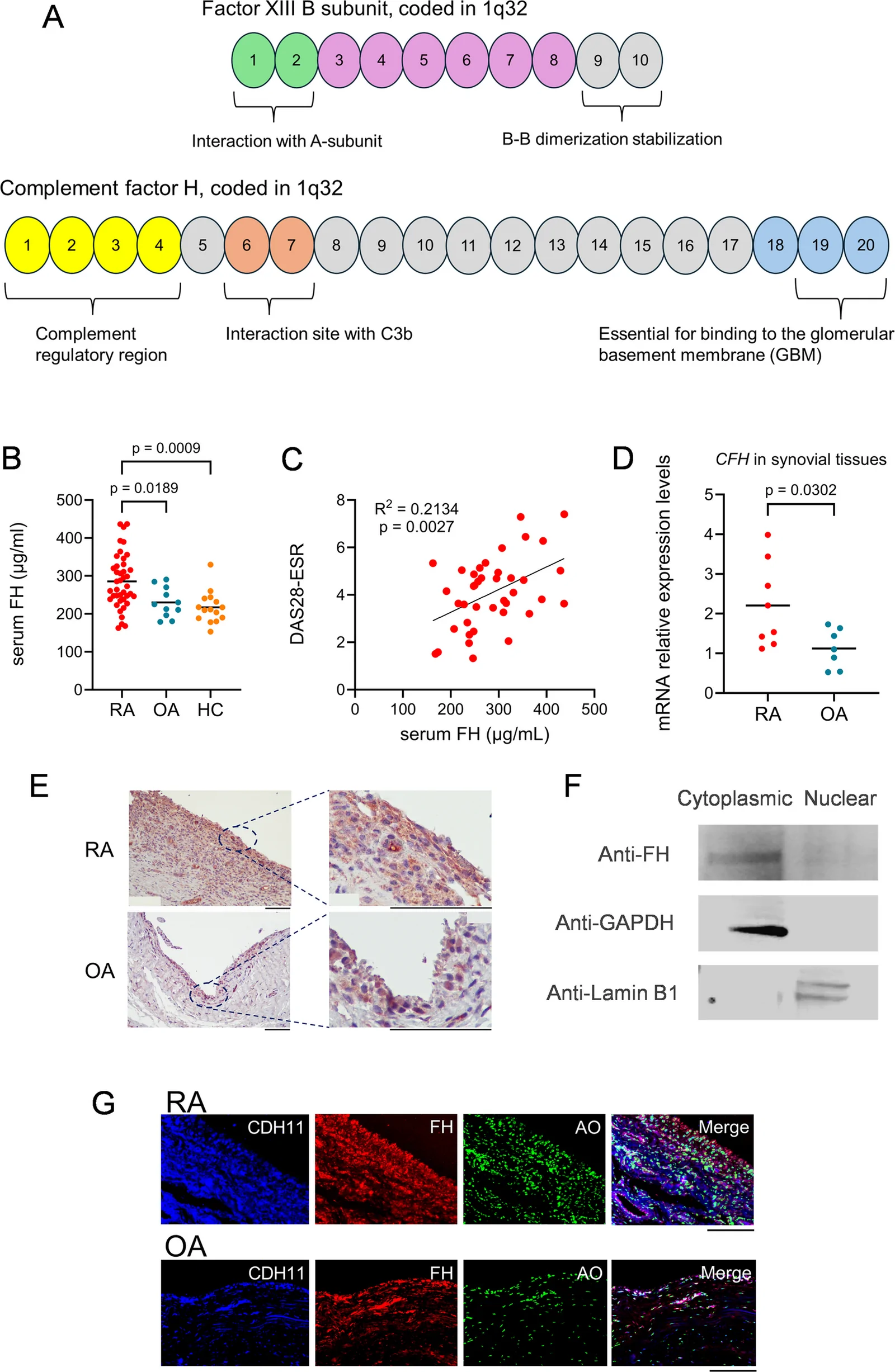
FH expression patterns in serum and synovial tissues. **A** Domain schematic diagrams of FXIII-B and FH. **B** Serum FH protein levels (µg/mL) measured with ELISA in RA (*n* = 41), OA (*n* = 11), and HC (*n* = 15). Black lines indicate the mean. Data were analyzed using Dunnett’s test. **C** Relationship between serum FH levels and RA disease activity (DAS28-ESR) (*n* = 41). Data were analyzed using Pearson’s correlation coefficient. A linear regression line was plotted to visualize the relationship. **D** *CFH* expression levels in whole synovial tissues of RA (*n* = 8) and OA (*n* = 7). Data were analyzed using Student’s *t*-test. **E** IHC analysis of synovial tissues (red, FH). Representative images are shown. **F** FH protein localization in FLS-derived cell line, MH7A. Western blotting analysis was performed on cytoplasmic and nuclear fractions. **G** Fluorescent IHC analysis of synovial tissues (blue, CDH11; red, FH; green, AO). Representative images are shown. Scale bar = 100 µm. FXIII-B, factor XIII B subunit; FH, factor H; CFH, complement factor H; RA, rheumatoid arthritis; OA, osteoarthritis; HC, healthy control; CDH11, cadherin-11; AO, acridine orange

RA is characterized by multilayered immunopathology, in which innate immunity, adaptive immunity, and the complement system (an interface between innate and adaptive immunity) are intimately involved [16, 17]. The complement system consists of activating components, such as C3 and C4, and inhibitory regulators, including complement factor H (FH; gene name *CFH*) [18]. FH is a key regulator of the alternative complement pathway in the circulation and extracellular environment, where it suppresses excessive complement activation through C3b inactivation [19, 20]. Similar to factor XIII B (FXIII-B; gene name *F13B*), FH and factor H-related proteins 1–5 (FHR1–5) are encoded within the chromosome 1q32 regulators of complement activation (RCA) gene cluster and are composed exclusively of Sushi domains, also known as complement control protein (CCP) domains (Fig. 1A) [19, 21, 22]. FXIII-B consists of 10 Sushi domains and functions as a carrier protein for the coagulation factor FXIII-A [23, 24]. And FH is composed of 20 Sushi domains [19]. Serum FH levels have been reported to be correlated with disease activity in RA [25]. Although this association suggests a potential contribution of FH to RA pathogenesis, its local functions within the synovium and its role in lesion development remain poorly understood.

In this study, we investigated the expression and function of intracellular FH using an in vitro transcribed messenger RNA (IVT mRNA)-based approach. We found that intracellular FH is tightly linked to TNF signaling and acts as an essential amplifier of TNF-driven inflammatory responses in RA-derived FLS. Specifically, FH enhances TNF-induced signaling outputs and coordinately promotes the expression of key pathogenic mediators, including GM-CSF, IL-8, and IL-6.

## Methods

### Study design

This study received approval from the Clinical Ethics Committees of Hiroshima University Hospital, Dohgo Spa Hospital, and the Ehime University Proteo-Science Center and Graduate School of Medicine (approval number: E2016-668-05; approval date: June 18, 2024). All procedures were conducted in accordance with the approved protocols. Serum and synovial tissues were obtained from patients with RA and non-RA controls (patients with osteoarthritis [OA] and healthy volunteers) who fulfilled the 1987 American College of Rheumatology classification criteria [26] and underwent total joint replacement after providing informed consent. Clinical information for each patient was retrieved from medical records.

### Enzyme-linked immunosorbent assay (ELISA)

Serum and culture supernatant levels of complement factor H (FH), granulocyte–macrophage colony-stimulating factor (GM-CSF), interleukin-8 (IL-8), and interleukin-6 (IL-6) were quantified using enzyme-linked immunosorbent assays (ELISA). Culture supernatants were collected 12–18 h after medium replacement, which was performed 6–12 h following IVT mRNA transfection. Human FH concentrations were measured using the Human Complement Factor H SimpleStep ELISA Kit (Abcam; Abcam, Cambridge, UK). Human GM-CSF, IL-8, and IL-6 levels were determined using the ELISA MAX Standard Set (BioLegend, San Diego, CA, USA). All assays were performed in duplicate in accordance with the manufacturer’s instructions. Absorbance was read at 450 nm with wavelength correction at 570 nm using a SpectraMax iD3 microplate reader (Molecular Devices, San Jose, CA, USA).

### Primary cultured and immortalized FLS preparation and culture

Primary human FLS were obtained from synovial tissues collected from patients with RA. All patients with RA from whom primary FLS were established had received treatment with conventional synthetic disease-modifying antirheumatic drugs (csDMARDs) only; none had been exposed to cytokine-targeting biologics or other cytokine inhibitors. In brief, synovial samples obtained during total joint replacement procedures were finely minced and digested in phosphate-buffered saline (PBS) containing 1 mg/mL collagenase/dispase (Roche, Mannheim, Germany) at 37 °C for 1 h, followed by filtration. For FLS isolation, the resulting cell suspensions were diluted and plated in culture dishes. During the early culture period, the medium was frequently replaced to remove non-adherent cells. Adherent FLS were expanded under subconfluent conditions and passaged at a 1:3 ratio. Cells were maintained in Dulbecco’s Modified Eagle Medium (DMEM; FUJIFILM Wako Pure Chemical Co., Osaka, Japan) supplemented with 10% fetal bovine serum (FBS; Gibco, Waltham, MA, USA) and penicillin/streptomycin (FUJIFILM Wako Pure Chemical Co.). FLS between passages 3 and 6 was used for experiments. Immortalized FLS derived from patients with RA (MH7A cells) were provided by the RIKEN BRC through the National BioResource Project of the MEXT (Tsukuba, Japan) and KISSEI Pharmaceutical Co., Ltd. (Matsumoto, Japan). MH7A cells were cultured in Roswell Park Memorial Institute (RPMI) 1640 medium (FUJIFILM Wako Pure Chemical Co.) supplemented with 10% FBS and penicillin/streptomycin.

For cytokine stimulation with recombinant human tumor necrosis factor-alpha (TNF, 50 ng/mL), interleukin-1-beta (IL-1β, 10 ng/mL), IL-6 (50 ng/mL), soluble IL-6 receptor alpha (sIL-6R, 50 ng/mL), and IL-17A (50 ng/mL) (Biolegend), cells were serum-starved in DMEM containing 0.1% FBS for 12 h before treatment. For neutralization experiments, the following antibodies (5 μg/mL) were applied 30 min before cytokine stimulation: anti-TNF antibody (mouse monoclonal antibody [mAb], clone MAB610; R&D Systems, Minneapolis, MN, USA), mouse IgG1 isotype control (mouse mAb, clone MAB002; R&D Systems), anti-FH antibody (rabbit polyclonal Ab [pAb]; Bioss Antibodies Inc., Woburn, MA, USA), and normal rabbit IgG (Cell Signaling Technology, Danvers, MA, USA). For pathway-inhibition experiments, the competitive nucleotide-binding oligomerization domain 2 (NOD2) antagonist GSK717 (MedChemExpress, Monmouth Junction, NJ, USA) was added 30 min before mRNA transfection. For endoplasmic reticulum (ER) stress experiments, tunicamycin (Cayman Chemical, Ann Arbor, MI, USA) was added as a positive control.

### Plasmid construction and transfection into MH7A cells

Full-length RelA (p65; *RELA*, NM_021975.4) was amplified using PCR and inserted into the pcDNA3 vector (Invitrogen, Carlsbad, CA, USA), as previously described [27]. Four expression plasmids based on the pcDNA3-A(124) vector [28] were used in this study: HA-tagged full-length *CFH*, HA-tagged full-length *F13B*, enhanced green fluorescent protein (EGFP), and an empty vector (mock). The plasmid encoding *F13B* (NM_001994.3) was prepared as previously described [29]. For the plasmid encoding *CFH* (NM_000186.4), the insert containing the HA-tagged *CFH* open reading frame was purchased from GenScript (Tokyo, Japan). MH7A cells were transfected with each plasmid using ScreenFectA plus (FUJIFILM Wako Pure Chemical Co.) according to the manufacturer’s protocol. For transfection in a 12-well plate, 0.02–0.5 μg of plasmid DNA per well was used. After transfection, cells were cultured under standard conditions for 24 h and harvested for subsequent analyses.

### Transfection of in vitro transcribed mRNA (IVT mRNA) into primary cultured FLS and MH7A cells

IVT mRNA was generated as previously described [30]. IVT mRNA was transfected into cells using Lipofectamine MessengerMAX (Invitrogen) according to the manufacturer’s instructions. Briefly, IVT mRNA was mixed with Lipofectamine reagent at a ratio of 1:3 (pmol mRNA: μL Lipofectamine) in serum-free RPMI medium and incubated for 20 min at 25 °C to allow complex formation. For transfection in a 12-well plate, 0.5 pmol of IVT mRNA was added per well. After transfection, cells were collected for subsequent analyses.

### RNA extraction and reverse transcription quantitative PCR (RT-qPCR)

Total RNA was isolated and purified from whole synovial tissues and cultured cells using TRIzol reagent (Life Technologies, Carlsbad, CA, USA) in combination with the Direct-zol RNA kit (Zymo Research, Irvine, CA, USA). Complementary DNA (cDNA) was synthesized from mRNA using the PrimeScript RT Reagent Kit with gDNA Eraser (Takara Bio, Kusatsu, Japan). RT-qPCR was performed with Brilliant II SYBR Green QPCR Master Mix (Agilent, Santa Clara, CA, USA) on a CFX Connect Real-Time PCR Detection System (Bio-Rad Laboratories, Hercules, CA, USA). The primers used in this study are listed in Additional file 1. Gene expression levels were normalized to the housekeeping gene *glyceraldehyde-3-phosphate dehydrogenase* (*GAPDH*).

### Next-generation sequencing (RNA-seq)

RNA sequencing library preparation, sequencing, read mapping, and gene expression analysis were performed by DNAFORM (Yokohama, Kanagawa, Japan). Total RNA was extracted from primary cultured FLS with the Direct-zol RNA kit (Zymo Research). The quality of total RNA was assessed using a Bioanalyzer (Agilent Technologies, Santa Clara, CA, USA) to confirm that the RNA integrity number (RIN) exceeded 7.0. Double-stranded cDNA libraries (RNA-seq libraries) were prepared using the SMART-Seq Stranded Kit (TaKaRa, catalog no. 634442) and the DNBSEQ MGIEasy Universal Library Conversion Kit (MGI Tech) according to the manufacturer’s instructions. RNA-seq libraries were sequenced using paired-end reads on a DNBSEQ-G400RS instrument (MGI Tech), generating 150-nucleotide reads for Read 1 and Read 2. For the RNA-seq dataset generated in Fig. 3A, raw sequencing reads were trimmed and quality filtered using Trim Galore! (version 0.6.7; https://github.com/FelixKrueger/TrimGalore), Trimmomatic (version 0.39; https://github.com/usadellab/Trimmomatic) and cutadapt (version 3.7; https://github.com/marcelm/cutadapt) software. Trimmed reads were mapped to the human GRCh38 reference genome using STAR (version 2.7.10a; https://github.com/alexdobin/STAR). Gene-level read counts were generated using featureCounts (version 2.0.1; https://subread.sourceforge.net/) with a modified Gene Transfer Format (GTF) annotation file in which all *CFH*-associated records had been removed prior to read counting. Consequently, *CFH* was excluded from the gene expression count matrix at the read-counting step, while read mapping was performed against the standard GRCh38 genome without the addition of CFH IVT mRNA or EGFP IVT mRNA sequences. Differentially expressed genes were identified using the DESeq2 package (version 1.20.0; https://bioconductor.org/packages/release/bioc/html/DESeq2.html). Genes showing greater relative fold changes were selected as candidate upregulated genes for the ontology analysis.

For the analysis presented in Additional file 10, we reanalyzed our previously reported RNA-seq dataset [31], which is deposited under accession number PRJNA915654, using a different analytical approach. Raw sequencing reads were trimmed and quality filtered using Trim Galore! (version 0.6.7), Trimmomatic (version 0.39) and cutadapt (version 3.7) software. Trimmed reads were mapped to the human GRCh38 reference genome using STAR (version 2.7.10a). Reads aligned to annotated genes were counted using featureCounts (version 2.0.1). Transcripts per million (TPM) values were calculated from mapped reads by normalization to total read counts and transcript length.

### Western blotting

Before sample preparation, MH7A cells and FLS were rinsed with PBS. The cells were then lysed in RIPA buffer (FUJIFILM Wako Pure Chemical Co.). After lysis, the samples were centrifuged at 15,000 × g for 5 min at 20–25 °C, and the resulting supernatants were collected. Protein lysates were separated on SuperSepAce 5–12% precast gels (FUJIFILM Wako Pure Chemical Co.) and subsequently transferred to polyvinylidene fluoride (PVDF) membranes. The membranes were probed using the following primary antibodies: anti-FH (rabbit pAb, Proteintech, Rosemont, IL, USA), anti-FXIII-B (rabbit pAb, Sigma-Aldrich, St Louis, MO, USA), anti-IκBα (rabbit pAb, Proteintech), anti-phospho-IκBα (Ser32/36) (rabbit mAb, clone 1G21, Proteintech), anti-receptor-interacting serine/threonine-protein kinase 2 (RIPK2) (rabbit pAb, Cusabio, Wuhan, China), anti-phospho-RIPK2 (S178) (rabbit pAb, Cusabio), anti-phospho-eIF-2α-Ser51 (rabbit mAb, clone 4N22, Sigma-Aldrich), anti- eIF-2α (rabbit pAb, Santa Cruz Biotechnology, Dallas, TX, USA), anti-HA-tag (mouse mAb, clone TANA2, MBL, Tokyo, Japan), anti-Lamin B1 (rabbit polyclonal, Santa Cruz Biotechnology), anti-GAPDH (goat polyclonal, R&D Systems), and anti-human β-actin antibody (mouse mAb, clone AC-15, Sigma-Aldrich). Following incubation with primary antibodies, the membranes were incubated with horseradish peroxidase (HRP)-conjugated secondary antibodies (donkey host; Jackson ImmunoResearch, West Grove, PA, USA). HRP activity was visualized using an ImageQuant LAS 500 system (Cytiva, Tokyo, Japan) with ECL Prime detection reagent (Cytiva).

For subcellular fractionation, nuclear and cytoplasmic extracts were prepared using NE-PER Nuclear and Cytoplasmic Extraction Reagents (Thermo Fisher Scientific, Waltham, MA, USA) according to the manufacturer’s instructions.

### Immunohistochemistry (IHC) staining for formalin-fixed paraffin-embedded (FFPE) synovial tissues

Formalin-fixed paraffin-embedded (FFPE) synovial tissue sections were deparaffinized and subjected to antigen retrieval in Tris-EDTA buffer (pH 9.0). For the enzyme-based method, sections were incubated with a primary antibody against FH (rabbit pAb, Proteintech). After washing, the sections were treated with biotin-conjugated secondary antibodies (Vector Laboratories, Burlingame, CA, USA), followed by alkaline phosphatase (AP)-conjugated streptavidin. Signals were visualized using the ImmPACT Vector Red AP Substrate (Vector Laboratories), and nuclei were counterstained with hematoxylin. For immunofluorescence staining, the specimens were incubated with primary antibodies against FH (rabbit pAb, Proteintech) and Cadherin-11 (CDH11) (goat pAb, R&D Systems). Alexa Fluor 405- or Alexa Fluor 647-conjugated secondary antibodies (Abcam) were used for detection, and nuclei were stained with acridine orange (Abcam). Fluorescence images were acquired using a BZ-X700 fluorescence microscope (Keyence).

### Immunofluorescence staining for MH7A cells

MH7A cells were seeded onto glass slides and transfected with IVT mRNA encoding EGFP, F13B-HA, or CFH-HA. Cells were fixed with acetone: methanol solution (1:1) for 20 min, washed, and blocked with 5% Blocking One-P (Nakalai Tesque, Kyoto, Japan). Primary antibodies were applied as follows: anti-p65 (rabbit mAb, clone D14E12, Cell Signaling Technology), anti-HA-tag (rabbit pAb, MBL), anti-EGFP (rabbit pAb, Proteintech), and normal rabbit IgG (Cell Signaling Technology). Alexa Fluor 647-conjugated secondary antibody (Abcam) was used for detection. Nuclei were counterstained with 4′,6-diamidino-2-phenylindole (DAPI). NF-κB translocation was assessed by acquiring fluorescence images using a Keyence BZ-X700 microscope and manually counting cells exhibiting predominant nuclear localization of p65 across multiple randomly selected fields.

### Luciferase assay for CFH promoter activity

For the luciferase assay, a reporter plasmid was constructed using custom gene products (GenScript). The pGL4.16 vector was purchased from Promega (Madison, WI, USA). The pGL4.16-hCFH promoter (pGL4.16-hCFHp) vector was generated by inserting the human CFH promoter region (− 632 to + 135 base pairs [bp] relative to the transcription start site [TSS]) into the pGL4.16 vector. MH7A cells were transfected with the reporter plasmid and a Renilla luciferase control vector (pRL-SV40), together with the pcDNA3-p65 expression vector, using ScreenFectA plus (FUJIFILM Wako Pure Chemical Co.) according to the manufacturer’s instructions. For transfection in 24-well plates, 0.1 μg of the pGL4.16 and 0.01 μg of the Renilla plasmids were added per well. After 24 h of transfection, cells were lysed, and luciferase activity was measured using the Dual-Glo Luciferase Assay System (Promega). Firefly luciferase activity was normalized to Renilla luciferase activity to obtain relative promoter activity.

### Co-immunoprecipitation (IP) assays

Co-IP assays were carried out with MH7A cells transfected with HA-tagged CFH IVT mRNA using Lipofectamine MessengerMAX (Invitrogen), as described previously. Cells were harvested and solubilized in lysis buffer containing 25 mM Tris/HCl pH 7.4, 150 mM NaCl, 1% NP-40, 1 mM ethylenediaminetetraacetic acid (EDTA), and 5% glycerol. Equal amounts of cell lysates were prepared for each condition, and 400 µg of protein per sample was incubated with an anti-HA-tag antibody (mouse mAb, clone TANA2, MBL) or mouse control IgG. Immune complexes were then captured using Dynabeads Protein G (Invitrogen) following the manufacturer’s protocol. The beads were washed with PBS, and bound proteins were eluted in sodium dodecyl sulfate (SDS) sample buffer. Western blotting was subsequently performed as described above using anti-FH antibody (rabbit pAb, Proteintech) and anti-NOD2 antibody (rabbit pAb, Invitrogen).

### Cell viability assay using calcein-AM

MH7A cells (5.0 × 10^3^ cells/well) were seeded into 96-well plates in RPMI medium supplemented with 10% FBS. After 24 h of culture, the cells were transfected with either mock IVT mRNA or CFH IVT mRNA, as described above. Cell viability was assessed using calcein-AM (Cell Counting Kit-F; Dojindo Laboratories, Kumamoto, Japan) at 24 or 96 h after transfection, following the manufacturer’s instructions. Calcein fluorescence was quantified using a SpectraMax iD3 plate reader (Molecular Devices, San Jose, CA, USA) with excitation and emission wavelengths of 485 and 525 nm, respectively.

### Transfection of small interfering RNA (siRNA) into MH7A cells

siRNA targeting *CFH* (FlexiTube siRNA, SI03095869) and the negative control siRNA (Allstar Negative Control siRNA) were purchased from Qiagen (Shanghai, China). For siRNA transfections, MH7A cells were treated with Lipofectamine RNAiMAX (Invitrogen, Carlsbad, CA, USA) according to the manufacturer’s protocol. Harvested cells and culture supernatants were used in subsequent experiments.

### Statistical analysis

Statistical differences between the two groups were estimated using an unpaired Student’s t-test. Differences between three or more groups were analyzed using a one-way ANOVA, followed by Dunnett’s post hoc test. Correlation between serum FH levels and clinical parameters was assessed using Pearson’s correlation coefficient. Data processing and analyses were performed using GraphPad Prism 11 (GraphPad Software Inc., La Jolla, CA, USA). Data are presented as the mean ± standard deviation (SD).

## Results

### Expression and localization of complement factor H in RA sera and synovial tissues

To establish the clinical and tissue relevance of FH in RA, we first quantified serum FH levels and examined FH expression and localization in synovial tissues. Serum FH concentrations were significantly higher in patients with RA than in those with OA or in healthy controls (Fig. 1B). Serum FH levels positively correlated with RA disease activity, as assessed by DAS28-ESR (Fig. 1C), indicating that serum FH may serve as a biomarker candidate for RA. Consistent with these findings, *CFH* gene expression in synovial tissue was also higher in RA than in OA samples (Fig. 1D). IHC analysis of synovial tissue also demonstrated that FH protein expression was higher in RA than in OA and showed intracellular staining patterns (Fig. 1E). Subcellular fractionation analysis further confirmed the presence of FH in the cytoplasmic fraction (Fig. 1F). These findings support the intracellular location of FH. FH staining largely overlapped with that of CDH11, a marker of FLS (Fig. 1G), suggesting that FH is predominantly expressed by FLS.

### FH expression in FLS is induced by TNF

**Fig. 2:**
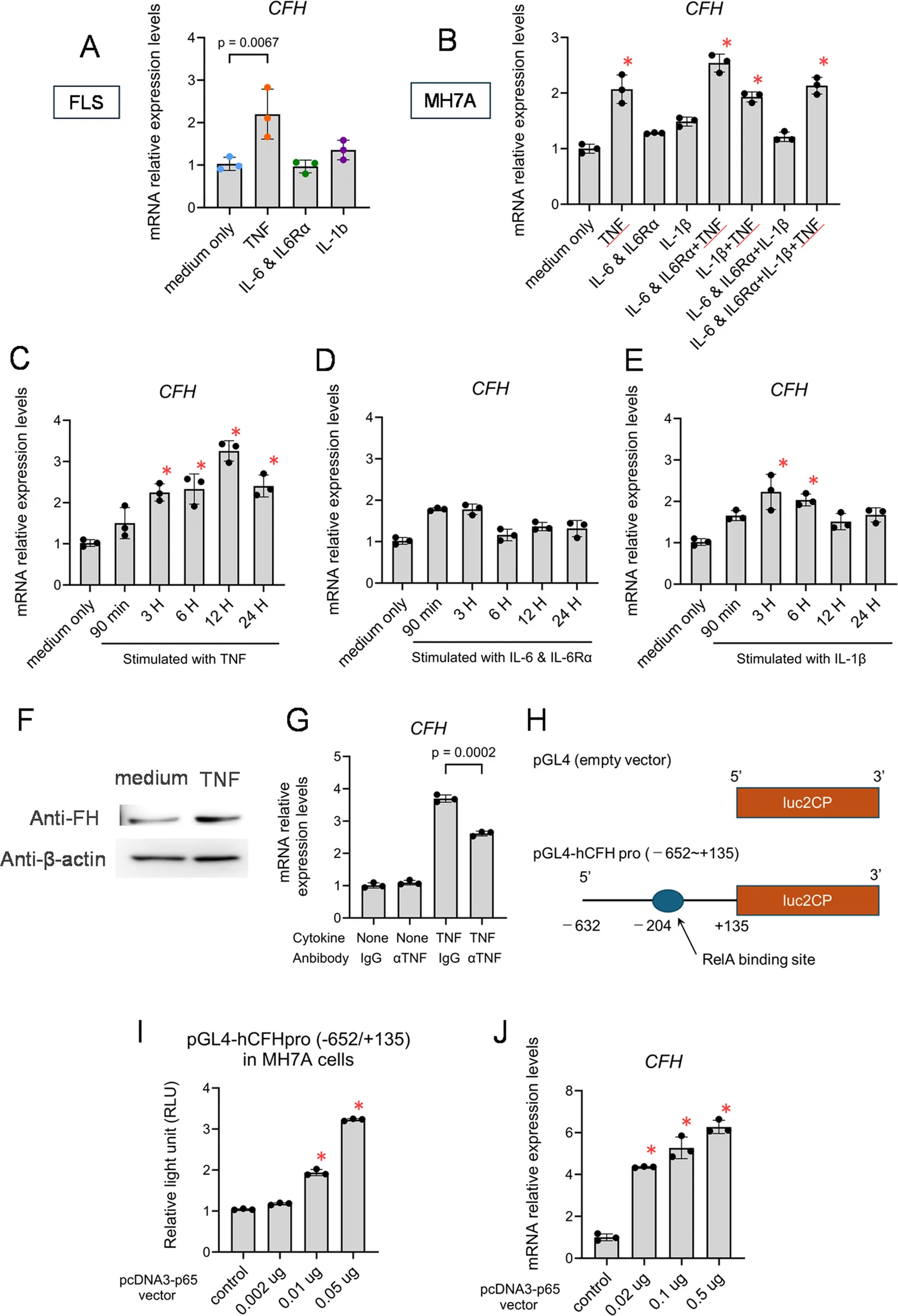
FH in fibroblast-like synoviocytes (FLS) is induced by TNF through the NF-κB pathway. **A** *CFH* expression levels in FLS were measured by RT-qPCR (*n* = 3, each from an individual subject). FLS was treated with various cytokines for 24 h. Data were analyzed using Dunnett’s test. **B**–**E**, **G**, **J** RT-qPCR analysis of *CFH* expression in MH7A cells. **B** *CFH* expression levels after 24 h of treatment with various cytokines (*n* = 3). Data were analyzed using Dunnett’s test (*p < 0.01). **C**–**E** *CFH* expression levels after treatment with each cytokine within 24 h (*n* = 3). Data were analyzed using Dunnett’s test (*p < 0.01). **F** FH expression in MH7A cells stimulated with TNF for 24 h was detected by Western blotting. **G** *CFH* expression levels after 12 h of co-treatment with TNF and anti-TNF neutralizing antibody (or control IgG) (*n* = 3, Student’s *t*-test). **H** Schematic diagrams of the human *CFH* promoter region. **I** Luciferase reporter assay for the *CFH* promoter region. Reporter and NF-κB (RELA/p65) expression plasmids (or control vector) were co-transfected into MH7A cells (*n* = 3). Data were analyzed using Dunnett’s test (*p < 0.01). **J** CFH expression in MH7A cells transfected with a p65 expression plasmid (or control vector) (*n* = 3). Data were analyzed using Dunnett’s test (*p < 0.01). FLS, fibroblast-like synoviocytes; TNF, tumor necrosis factor; IL, interleukin; RELA, v-rel avian reticuloendotheliosis viral oncogene homolog A

Next, we cultured primary FLS and the FLS-derived cell line MH7A and stimulated these samples with various cytokines to identify the extracellular stimulation that regulates endogenous *CFH* gene expression. In FLS, *CFH* expression was markedly increased following TNF stimulation, whereas IL-6 induced little or no change (Fig. 2A). IL-1β did not significantly affect *CFH* expression. Similar results were observed in MH7A cells. Notably, even when multiple cytokines were applied simultaneously, upregulation of *CFH* expression required the presence of TNF in the culture supernatant (Fig. 2B). Time-course analysis revealed that TNF-induced *CFH* expression peaked at 12 h (Fig. 2C), whereas IL-6 again exerted minimal effects (Fig. 2D). IL-1β induced a transient increase in *CFH* expression that peaked at 3 h, while TNF stimulation resulted in a stronger and more sustained induction of *CFH* expression over time (Fig. 2E). TNF-dependent upregulation of intracellular FH protein was further confirmed by immunoblot analysis of cell lysates (Fig. 2F). Moreover, neutralization of TNF using an anti-TNF antibody suppressed TNF-induced *CFH* expression (Fig. 2G). Collectively, these findings demonstrate that TNF is the predominant inducer of *CFH* expression in FLS. In contrast, expression of *F13B*, another gene encoding a protein composed exclusively of Sushi domains, was suppressed by TNF stimulation (Additional file 2A).

NF-κB is a major downstream mediator of TNF signaling; therefore, we examined whether NF-κB directly regulates *CFH* transcription. In silico analysis of the *CFH* promoter region using the JASPAR database (http://jaspar2014.genereg.net; accessed May 18, 2024) identified a putative NF-κB-binding site, including RelA (p65), located approximately 200 bp upstream of the transcription start site (Fig. 2H). Consistent with these findings, a luciferase reporter assay demonstrated that overexpression of p65 significantly activated the CFH promoter region (Fig. 2I). Furthermore, exogenous overexpression of p65 in MH7A cells resulted in increased endogenous *CFH* expression levels (Fig. 2J). Thus, TNF–NF-κB signaling is the predominant driver of CFH induction in FLS.

### Functional impact of intracellular FH on inflammatory gene expression in FLS

**Fig. 3:**
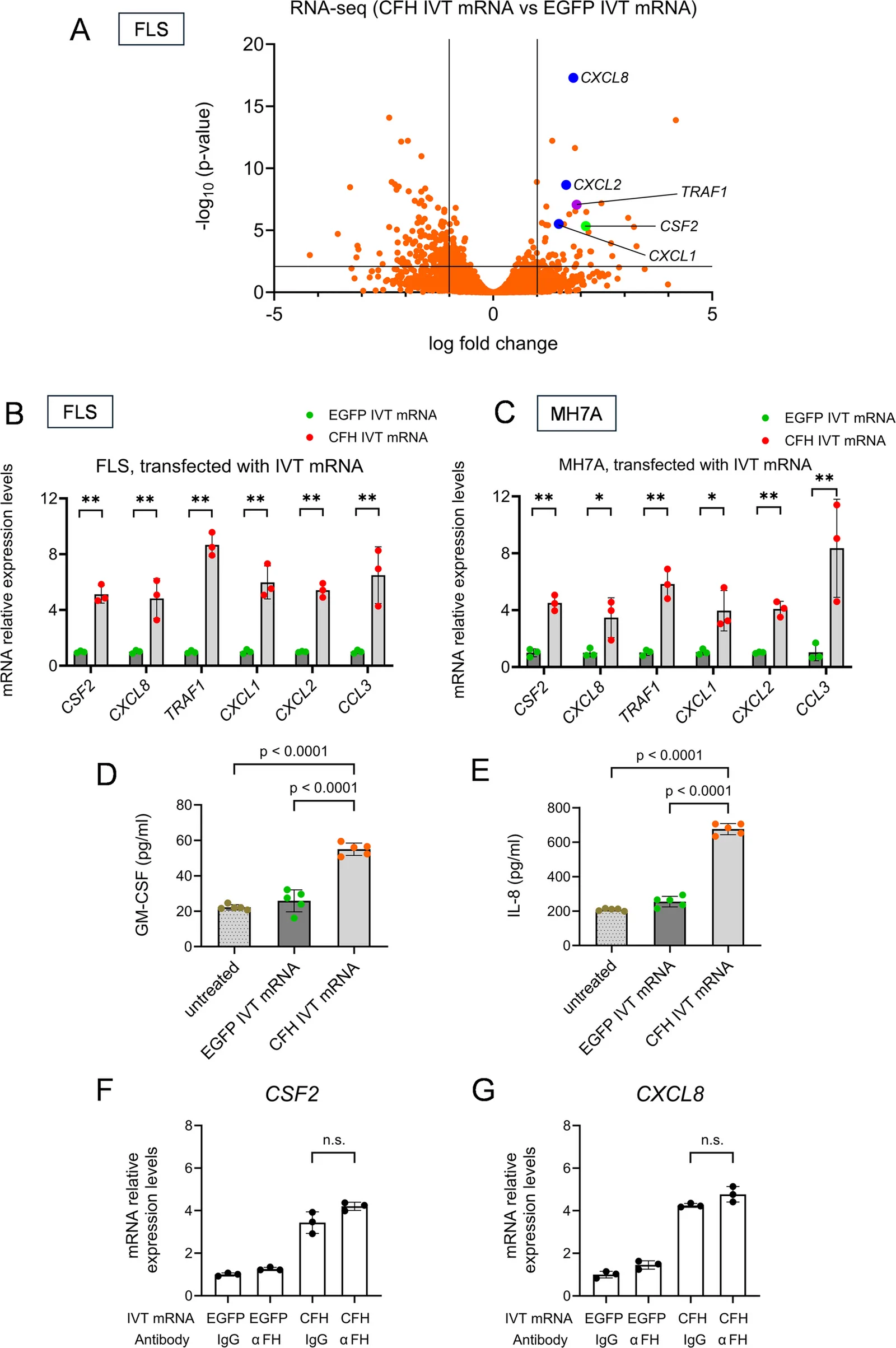
Several mediators, including GM-CSF and IL-8, are induced by CFH IVTmRNA in FLS. **A** RNA-seq analysis of FH-induced gene expression changes in primary cultured FLS. RNA from FLS transfected with CFH IVT mRNA or EGFP IVT mRNA (negative control) was collected 24 h after transfection and analyzed using next-generation RNA sequencing (RNA-seq) (*n* = 4, each from an individual subject). **B**, **C** Expression levels of *CSF2*, *CXCL8*, *TRAF1*, *CXCL1*, *CXCL2*, and *CCL3* in CFH IVT mRNA- or EGFP IVT mRNA-transfected cells were measured by RT-qPCR (*n* = 3). Data were analyzed using Student’s *t*-test (*p < 0.05, **p < 0.01). **B** FLS. **C** MH7A cells. **D**, **E** GM-CSF and IL-8 concentrations in the culture supernatants of MH7A cells transfected with CFH IVT mRNA or EGFP IVT mRNA were detected using ELISA (*n* = 5). Data were analyzed using Dunnett’s test. **D** GM-CSF. **E** IL-8. **F**, **G** RT-qPCR analysis of *CSF2* and *CXCL8* expression in MH7A cells transfected with CFH IVT mRNA or EGFP IVT mRNA and treated with control IgG or anti-FH antibody (*n* = 3, Student’s *t*-test). CSF2, colony stimulating factor 2; CXCL8, C-X-C motif chemokine ligand 8; TRAF1, TNF receptor-associated factor 1; CXCL1, C-X-C motif chemokine ligand 1; CXCL2, C-X-C motif chemokine ligand 2; CCL3, C-C motif chemokine ligand 3; GM-CSF, granulocyte–macrophage colony-stimulating factor; IgG, immunoglobulin G

We subsequently investigated the function of FH within FLS. In 2023, we reported the role of FXIII-B, another Sushi domain-only protein, in FLS [29]. In that previous study, IVT mRNA-mediated expression of FXIII-B enhanced the expression of multiple chemokines, including IL-8 (gene name *C-X-C Motif Chemokine Ligand 8* [*CXCL8*]), *CXCL1*, *CXCL2*, *C-C Motif Chemokine Ligand 3 (CCL3*), and *TNF Receptor Associated Factor 1* (*TRAF1*) in FLS [29]. Because FXIII-B and FH are secreted proteins, plasmid-driven overexpression resulted in limited intracellular retention and did not produce detectable downstream transcriptional changes, making functional analysis technically challenging (Additional file 3A, 3B and Additional file 4). In contrast, IVT mRNA-mediated expression enables efficient intracellular detection of these proteins, thereby permitting functional assessment (Additional file 3C–G). We also examined whether IVT mRNA-mediated expression affected ER stress markers and found no significant changes (Additional file 5A–C). Using this approach, we introduced CFH IVT mRNA into primary cultured FLS and performed transcriptomic analysis using RNA sequencing (RNA-seq) (Fig. 3A). Increased intracellular FH significantly enhanced the expression of *CSF2*, which encodes GM-CSF. Similar to FXIII-B, intracellular FH also upregulated *TRAF1* and multiple chemokines, most prominently *CXCL8*. Gene ontology analysis of the upregulated genes, performed using DAVID Bioinformatics Resources v2025 (https://david.ncifcrf.gov/, accessed on July 7, 2026) revealed significant enrichment of cytokine–cytokine receptor interactions, TNF signaling pathways, and RA-related pathways (Additional file 6). These gene expression changes were validated by RT-qPCR in primary FLS (Fig. 3B), and comparable results were obtained in MH7A cells, a FLS-derived cell line (Fig. 3C). At the protein level, enzyme-linked immunosorbent assay (ELISA) analysis of culture supernatants confirmed increased secretion of GM-CSF and IL-8 following CFH IVT mRNA transfection (Fig. 3D, E). Notably, the enhanced expression of *CSF2* was observed following the introduction of F13B IVT mRNA in the present study (Additional file 2B), whereas the induction of *CXCL8* has been previously reported [29]. These findings indicate that Sushi domain-only proteins, including FXIII-B and FH, could broadly promote the expression of multiple inflammation-related genes in FLS. Additional experiments showing that anti-FH antibody treatment did not suppress these responses further supported the functional contribution of intracellular, rather than extracellular, FH (Fig. 3F, G).

### Intracellular FH promotes nuclear translocation of p65 through RIPK2 and IκBα phosphorylation

**Fig. 4:**
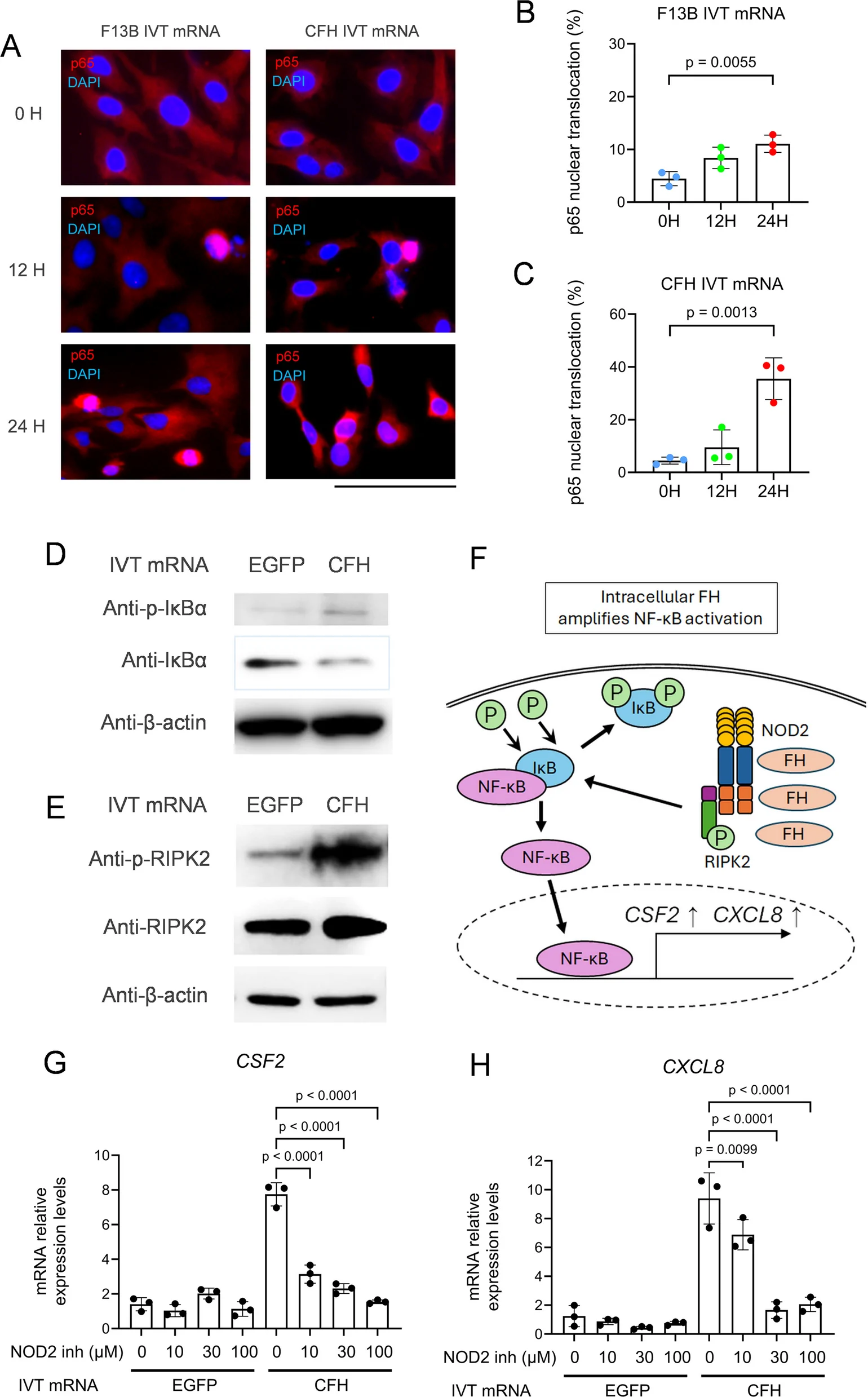
Intracellular FH activates the NOD2/RIPK2/NF-κB signaling cascade. **A**–**C** Immunocytochemical analysis of MH7A cells expressing FXIII-B or FH following transfection with F13B IVT mRNA or CFH IVT mRNA, respectively, at 0, 12, and 24 h. **A** Cells were stained with anti-p65 antibody, followed by Alexa Fluor 647-conjugated secondary antibody and DAPI (red, p65; blue, DAPI). Representative images are shown. Scale bar = 100 µm. **B**, **C** Quantification of p65 nuclear translocation in a time-dependent manner (*n* = 3, Dunnett’s test). **B** F13B IVT mRNA. **C** CFH IVT mRNA. **D**, **E** Western blotting analyses for MH7A cell lysates, which were transfected with CFH IVT mRNA or EGFP IVT mRNA and harvested at 1 h. **D** IκBα and phosphorylated IκBα. **E** RIPK2 and phosphorylated RIPK2. **F** Schematic diagram of intracellular FH-mediated activation of the NOD2/RIPK2/NF-κB axis. **G**, **H** RT-qPCR analysis for MH7A cells treated with NOD2 inhibitor (GSK717, a competitive antagonist) and subsequently transfected with CFH IVT mRNA or EGFP IVT mRNA for 24 h (*n* = 3, Dunnett’s test). **G** *CSF2* expression levels. **H** *CXCL8* expression levels. F13B, factor XIII B; DAPI, 4′,6-diamidino-2-phenylindole; IκBα, inhibitor of kappa B alpha; RIPK2, receptor-interacting serine/threonine-protein kinase 2

NF-κB is a central transcription factor that regulates multiple inflammatory genes induced by intracellular FH, including *CSF2*, *CXCL8*, *CXCL1*, and *CXCL2* [32–34]. Fluorescence immunocytochemical analysis revealed that MH7A cells transfected with CFH IVT mRNA exhibited a time-dependent increase in the proportion of cells showing nuclear localization of p65 (Fig. 4A–C). A similar trend was observed following transfection with F13B IVT mRNA, suggesting that this effect represents a shared intracellular functional feature of Sushi domain-only proteins. In addition, nuclear translocation of p65 occurred more frequently in FH-expressing cells than in FXIII-B-expressing cells. We subsequently examined phosphorylation events by immunoblot analysis to elucidate the intracellular signaling pathway associated with FH. Introduction of CFH IVT mRNA induced phosphorylation of IκBα (Fig. 4D) and increased phosphorylation of RIPK2, an upstream regulator of NF-κB signaling (Fig. 4E). Among known pattern-recognition receptors, NOD1 and NOD2 signal through RIPK2 to activate NF-κB [35] (Fig. 4F). We treated cells with GSK717, a competitive NOD2 antagonist, to probe the contribution of NOD2 signaling to intracellular FH-mediated inflammatory gene induction [36]. In the absence of CFH IVT mRNA transfection, GSK717 did not significantly alter the expression of *CSF2* or *CXCL8*. In contrast, the enhanced expression of these genes induced by intracellular FH was markedly suppressed by GSK717 treatment (Fig. 4G, H). Co-IP analysis showed endogenous NOD2 was detected in anti-HA immunoprecipitates from MH7A cells expressing HA-tagged FH, supporting the possibility that NOD2 is present in intracellular FH-containing protein complexes (Additional file 7). Collectively, these results suggest that intracellular FH promotes downstream gene expression via a NOD2/RIPK2/NF-κB signaling axis (Fig. 4F).

We subsequently examined whether TRAF1 is regulated through a similar FH-dependent pathway. Consistent with other FH-induced genes, treatment with the NOD2 inhibitor GSK717 significantly suppressed FH-induced TRAF1 expression (Additional file 8A). TRAF1 has been implicated in cell survival signaling [37], and CFH IVT mRNA transfection increased MH7A cell viability (Additional file 8B), whereas siRNA-mediated knockdown of *CFH* reduced cell viability (Additional file 8C and 8D). Collectively, these results indicate that intracellular FH contributes to FLS proliferation, at least in part, through TRAF1.

### TNF additively enhances RA-related gene expression together with intracellular FH

**Fig. 5:**
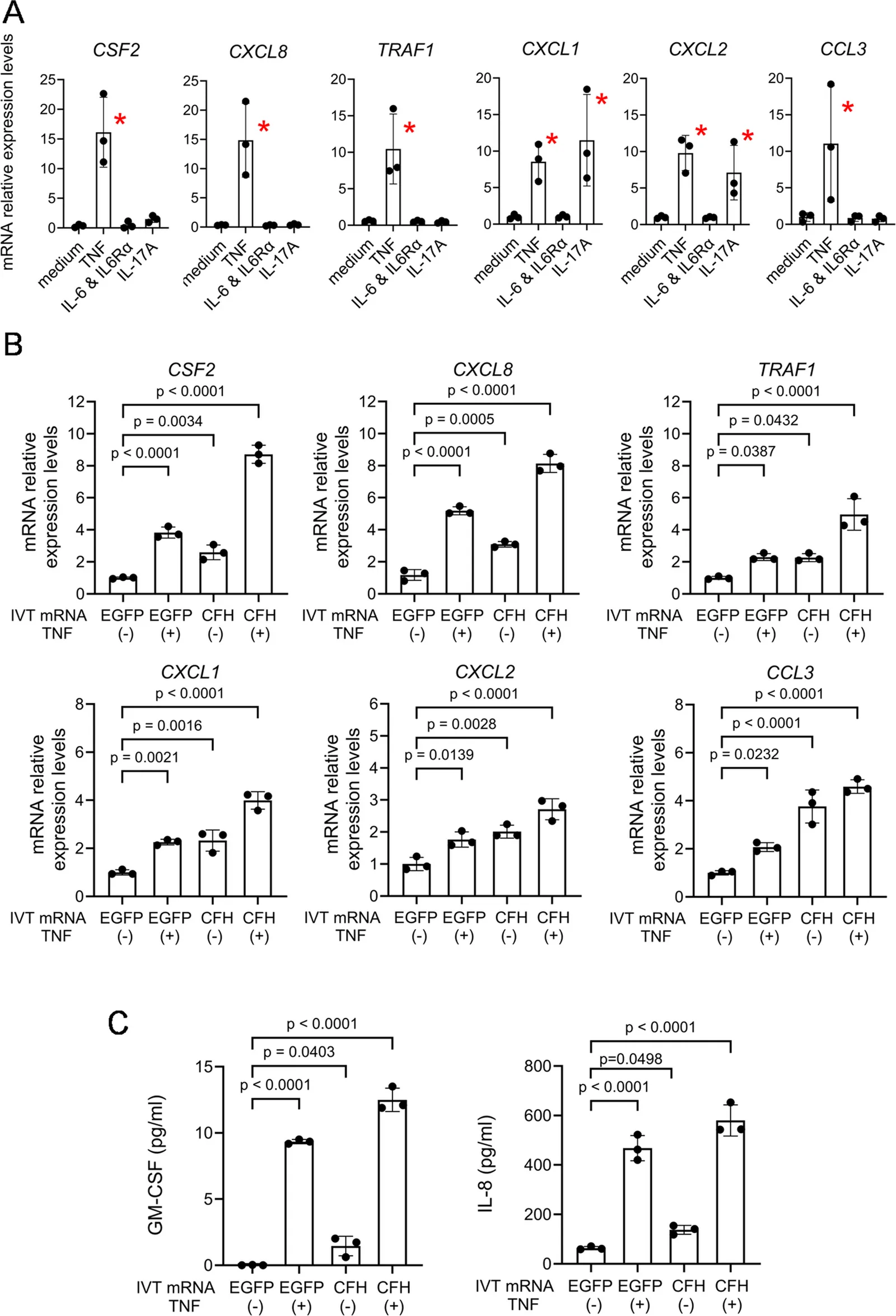
TNF additively enhances FH-induced inflammatory gene expression. **A** RT-qPCR analysis for expression levels of various factors (e.g., *CSF2*, *CXCL8*, *TRAF1*, *CXCL1*, *CXCL2*, and *CCL3*) in FLS stimulated with cytokines for 24 h (*n* = 3, each from an individual subject). Data were analyzed using Dunnett’s test (*p < 0.05). **B** RT-qPCR analysis of the expression levels of these various factors in MH7A cells following co-treatment with CFH IVT mRNA (or EGFP IVT mRNA) and/or TNF (*n* = 3, Dunnett’s test). Induction of IVT mRNA was performed for 12 h, after which TNF was added for an additional 12 h. **C** GM-CSF and IL-8 concentrations in the culture supernatants of MH7A cells transfected with CFH IVT mRNA (or EGFP IVT mRNA) and/or TNF were measured using ELISA (*n* = 3, Dunnett’s test)

**Fig. 6:**
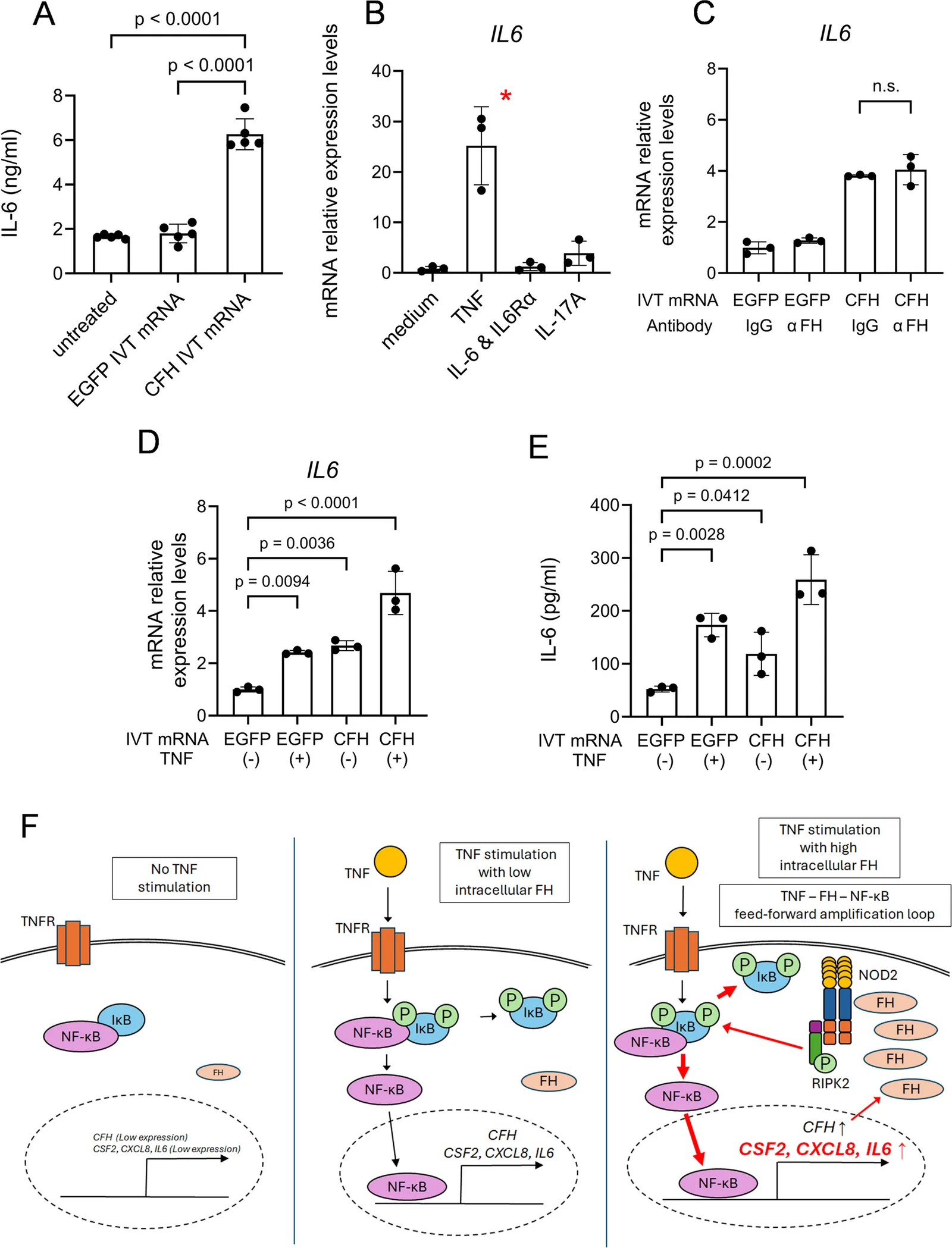
IL-6 is also additively induced by TNF and intracellular FH. **A** IL-6 concentrations in the culture supernatants of MH7A cells transfected with CFH IVT mRNA or EGFP IVT mRNA were detected using ELISA (*n* = 5). **B** RT-qPCR analysis for expression levels of *IL6* in FLS stimulated with cytokines for 24 h (*n* = 3, each from an individual subject, *p < 0.05). **C** RT-qPCR analysis of *IL6* expression in MH7A cells transfected with CFH IVT mRNA or EGFP IVT mRNA and treated with control IgG or anti-FH antibody (*n* = 3, Student’s *t*-test). **D** RT-qPCR analysis of the expression levels of *IL6* in MH7A cells following co-treatment with CFH IVT mRNA (or EGFP IVT mRNA) and/or TNF (*n* = 3). Induction of IVT mRNA was performed for 12 h, after which TNF was added for an additional 12 h. **E** IL-6 concentrations in the culture supernatants of MH7A cells transfected with CFH IVT mRNA (or EGFP IVT mRNA) and/or TNF were detected using ELISA (*n* = 3). **A**, **B**, **D**, **E** Data were analyzed using Dunnett’s test. **F** Schematic model illustrating the additive activation of NF-κB signaling by TNF and intracellular FH. Under TNF stimulation and high intracellular FH levels, intracellular FH enhances NOD2–RIPK2-mediated NF-κB activation, forming a feed-forward amplification loop that amplifies expression of NF-κB target genes such as *CSF2*, *CXCL8*, and *IL6*

We next analyzed expression levels of six candidate genes (*CSF2*, *CXCL8*, *TRAF1*, *CXCL1*, *CXCL2*, and *CCL3*) regulated by intracellular FH in primary cultured FLS under cytokine stimulation. All of these genes were upregulated by TNF stimulation (Fig. 5A). Given that these genes were induced by TNF and FH, we hypothesized that the two factors cooperatively regulate their expression. We tested this hypothesis by treating MH7A cells with TNF and CFH IVT mRNA in parallel. As shown in Fig. 5B, combined stimulation with TNF and FH led to additive increases in the expression of all six genes. At the protein level, ELISA confirmed additive increases in GM-CSF and IL-8 concentrations in the culture supernatants of MH7A cells following combined stimulation with TNF and FH (Fig. 5C). Although IL-6 did not reach statistical significance in the RNA-seq analysis (Fig. 3A), TNF stimulation and CFH IVT mRNA transfection induced IL-6 expression, as confirmed by ELISA and RT-qPCR (Fig. 6A, B). Treatment with an anti-FH antibody, which targets extracellular FH, did not suppress IL6 expression induced by CFH IVT mRNA transfection (Fig. 6C). Furthermore, combined stimulation with TNF and intracellular FH resulted in additive increases in *IL6* expression (Fig. 6D), with corresponding increases in IL-6 protein levels in the culture supernatants (Fig. 6E).

As illustrated in Fig. 6F, TNF activates NF-κB signaling, and intracellular FH promotes the same downstream genes through the NOD2/RIPK2/NF-κB axis. Combined stimulation resulted in additive regulation of downstream genes, including *CSF2*, *CXCL8*, *IL6*, and other chemokines.

### Concomitant inhibition of TNF and intracellular FH suppresses cytokine gene expression

**Fig. 7:**
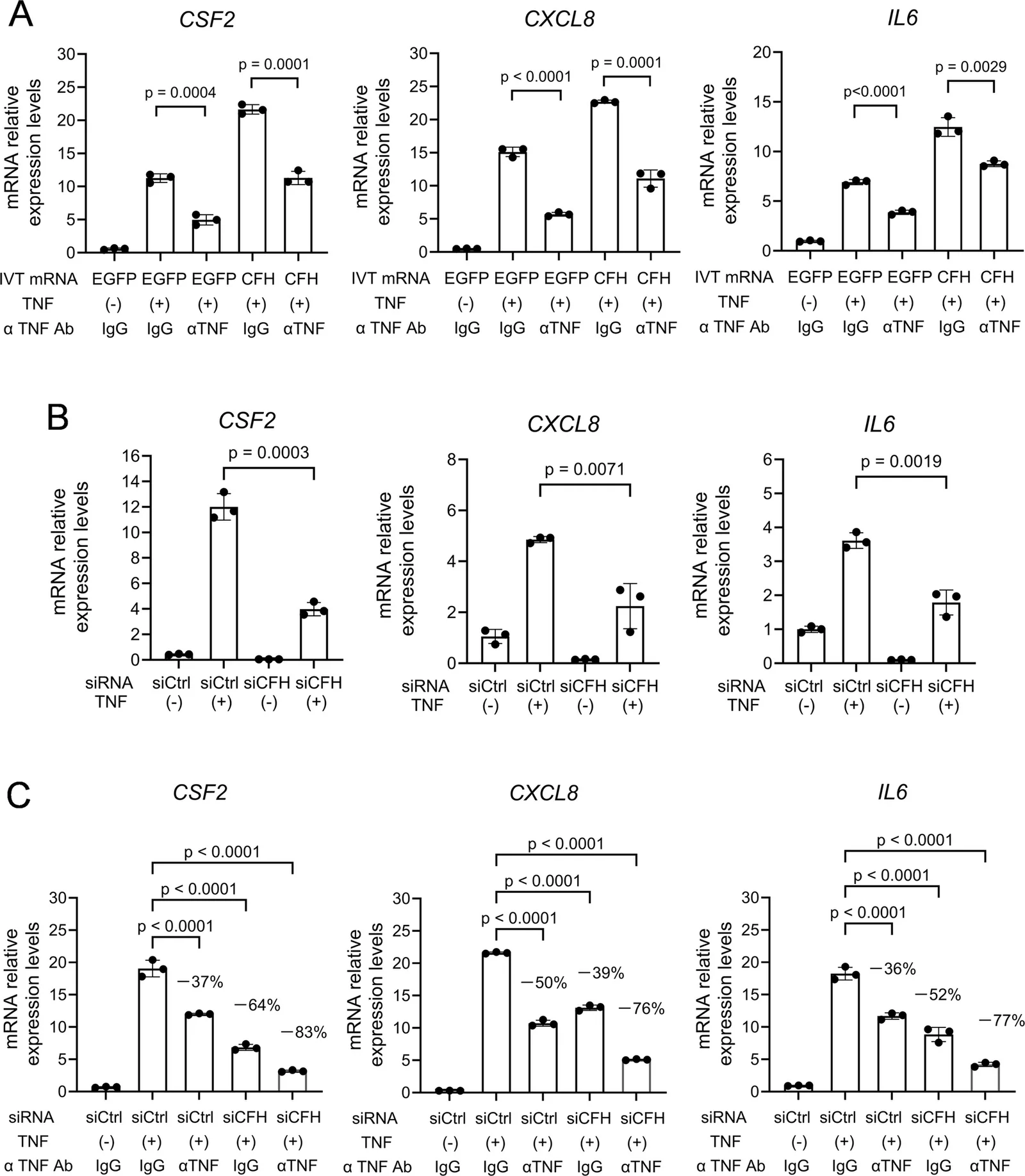
Suppression of TNF- and FH-induced cytokine expression by TNF neutralization and *CFH* knockdown. **A**–**C** RT-qPCR analysis of the expression levels of three cytokines (*CSF2*, *CXCL8*, and *IL6*) in MH7A cells (*n* = 3). Data were analyzed using Dunnett’s test. **A** Cells treated with TNF, CFH IVT mRNA (or EGFP IVT mRNA), and/or an anti-TNF antibody. IVT mRNA was transfected for 10 h, after which neutralization was performed, followed by the addition of TNF for 12 h. **B** Cells treated with TNF and 10 nM siRNA against *CFH* (siCFH) or control siRNA (siCtrl). siRNA induction was performed for 60 h, after which TNF was added for 12 h. **C** Cells treated with TNF and siCFH (or siCtrl) and/or an anti-TNF antibody. siRNA induction was performed for 60 h, after which neutralization was carried out, followed by the addition of TNF for 12 h

GM-CSF, IL-8, and IL-6 are key soluble mediators involved in RA pathogenesis. We assessed the suppression of these TNF-induced cytokines by examining the effects of TNF neutralization and *CFH* knockdown using a neutralizing antibody and siRNA, respectively, in TNF-stimulated cells. Treatment with a TNF-neutralizing antibody moderately suppressed the expression of *CSF2*, *CXCL8*, and *IL6* in cells treated with TNF and CFH IVT mRNA (Fig. 7A). In contrast, treatment with an anti-FH antibody did not alter the expression of these genes (Additional file 9A–C), indicating that extracellular FH does not contribute to the regulation of these cytokines. Subsequently, we assessed whether *CFH*-targeting siRNA could suppress cytokine gene expression in cells stimulated with TNF alone. Expression of *CSF2*, *CXCL8*, and *IL6* was moderately reduced following *CFH* knockdown (Fig. 7B), indicating that endogenous intracellular FH induced by TNF contributes to the regulation of these three RA-related mediators. Finally, by combining TNF neutralization with *CFH*-targeting siRNA, we observed the additive suppression of all three cytokine genes (Fig. 7C). Together, these findings suggest that inflammatory responses additively amplified by TNF signaling and TNF-induced intracellular FH can be effectively attenuated by the concomitant inhibition of TNF and this intracellular pathway.

## Discussion

In this study, using IVT mRNA technology, we identified a novel mechanism by which intracellular FH amplifies TNF-dependent inflammatory responses in FLS through the NOD2/RIPK2/NF-κB signaling axis. Although serum FH has been reported as a biomarker correlating with RA disease activity, its local roles and cell-intrinsic functions in the synovium, particularly in FLS, have remained largely uncharacterized. Our findings demonstrate that endogenous intracellular FH expression is induced by TNF stimulation and that intracellular FH cooperates with TNF to additively enhance the expression of pathogenic cytokines, including GM-CSF, IL-8, and IL-6. These results indicate that TNF-driven inflammation in synovial tissue may establish a feed-forward amplification loop via TNF-induced intracellular FH, thereby contributing to the chronicity of synovitis. As far as we know, this study is the first to quantitatively demonstrate an inflammation-amplifying function of intracellular FH.

As noted above, *F13B* encodes the protein FXIII-B, which is composed of 10 Sushi domains [20]. In 2023, we reported the expression pattern of FXIII-B in synovial tissue and its function in FLS [29]. In that study, we demonstrated that overexpression of FXIII-B increased the expression of multiple chemokines, including *CXCL8*, as well as *TRAF1*. In this study, we examined FH, a protein composed of 20 Sushi domains [19], and characterized its expression pattern in synovial tissue as well as its intracellular function in FLS. Although FH exhibited somewhat stronger activity than FXIII-B, both Sushi domain-containing proteins were found to promote nuclear translocation of cytoplasmic NF-κB. Accordingly, we demonstrated that this family of Sushi domain-only proteins contributes to enhanced downstream expression of six RA-associated genes, including *CSF2*, *CXCL8*, *TRAF1*, *CXCL1*, *CXCL2*, and *CCL3*.

Although FXIII-B and FH exhibit similar intracellular functions in FLS, their patterns of TNF-induced expression differ strikingly between the *F13B* and *CFH* genes. Upon TNF stimulation, the direction of gene expression change was entirely opposite between *F13B* and *CFH.* Although the underlying mechanism remains unclear, our data show that TNF stimulation suppresses *F13B* expression. These findings suggest that intracellular FXIII-B does not participate in a TNF-driven feed-forward amplification loop, in contrast to intracellular FH. Accordingly, we did not further investigate the relationship between TNF and intracellular FXIII-B in this study. Moreover, similar to FXIII-B and FH, FHR1–5 (gene names *CFHR1–CFHR5*, respectively) are composed exclusively of Sushi domains (CCP domains). RNA-seq analysis demonstrated that these family members are minimally or not expressed in FLS compared with *CFH* (Additional file 10). For this reason, we did not further investigate FHR proteins in this study. Regarding TNF-mediated regulation, TNF-induced upregulation of *CFH* has been previously reported by Jia et al. [25], consistent with our observations. Notably, Jia et al. reported that exogenously added soluble FH attenuated TNF-induced inflammatory responses in RA-derived cells, suggesting an anti-inflammatory effect of extracellular FH. In contrast, our findings indicate that intracellular FH amplifies inflammatory signaling in FLS. These observations raise the possibility that FH exerts compartment-dependent functions according to its localization. In addition, differences in FH composition and cell-type-specific responses may further contribute to the diverse functional roles of FH. Therefore, selectively suppressing intracellular FH within the synovium may represent a rational strategy to inhibit the amplification of pathogenic inflammation while preserving potentially protective extracellular FH. However, because our functional analyses were performed primarily in FLS, the therapeutic relevance of intracellular FH is currently best supported in FLS, whereas its role in other cell types remains unclear. We further demonstrate that NF-κB regulates *CFH* transcription via the promoter region, a finding that has not been previously reported. Although IL-6-mediated positive feedback has been reported under certain pathological conditions, its contribution appears to be context- and cell type-dependent [38, 39]. In our experimental setting, IL-6 alone or IL-6 plus sIL-6R induced little or no *CFH* expression, whereas TNF robustly induced CFH, suggesting that TNF is the predominant upstream driver in this FLS system.

We investigated the function of intracellular FH using IVT mRNA technology. To date, the only published study examining intracellular FH function is that by Daugan et al. in renal cell carcinoma, in which siRNA-mediated *CFH* knockdown revealed that intracellular FH (int-FH) contributes to transcriptional responses, maintenance of cellular homeostasis, and cell proliferation independently of the complement system [40]. Although the cell types differ, several functional similarities of intracellular FH were observed between their findings and our results in FLS.

NOD2 is classically activated by muramyl dipeptide (MDP), a component of bacterial peptidoglycan [41]. Apart from genetic mutations in NOD2, such as those observed in Blau syndrome, reports of NOD2 activation by endogenous factors remain limited [42, 43]. In this study, we demonstrate that intracellular FH, despite being an endogenous protein, activates the NOD2/RIPK2/NF-κB axis and amplifies TNF-driven inflammatory transcriptional outputs, thereby enhancing the expression of inflammation-related mediators. Together with the TNF-dependent induction of *CFH*, these findings support a TNF–intracellular FH feed-forward amplification loop possibly contributing to persistent inflammation and therapeutic resistance. Given that TNF and intracellular FH together enhance the expression of multiple RA-associated soluble mediators, including GM-CSF, IL-8, and IL-6, this intracellular FH–NOD2/RIPK2/NF-κB axis represents a potential therapeutic target in RA. One potential approach to disrupt this amplification is to suppress NOD2 signaling activated by intracellular FH. Consistent with this possibility, pharmacological inhibition of NOD2 may attenuate FH-dependent inflammatory responses. However, Nod2-deficient mice exhibit increased susceptibility to infection and metabolic abnormalities, including hepatic steatosis [44, 45], which raises concerns regarding systemic NOD2 inhibition. Thus, a safer and potentially more effective therapeutic strategy for RA may involve selectively suppressing intracellular FH levels locally within the synovium. In our in vitro analyses, TNF neutralization alone partially suppressed CFH expression in FLS but was insufficient to adequately suppress downstream mediators, including GM-CSF, IL-8, and IL-6. These findings suggest that TNF blockade by itself is insufficient to achieve robust, simultaneous suppression of multiple pathogenic mediators. In contrast, combining *CFH*-targeting siRNA with an anti-TNF neutralizing antibody resulted in additive and substantial inhibition of these inflammatory mediators. These results indicate that combinatorial targeting of TNF signaling and intracellular FH can effectively suppress this inflammatory amplification mechanism. Furthermore, because the majority of FH is produced in the liver and secreted into the circulation, selectively suppressing intracellular FH within synovial tissue is unlikely to markedly increase the risk of FH deficiency-associated disorders, such as atypical hemolytic uremic syndrome (aHUS) [46]. Collectively, these findings provide a rationale for selectively targeting intracellular FH within the synovium through intra-articular delivery of nucleic acid-based therapeutics as a potential treatment approach for RA.

## Conclusions

Overall, FH serves as a potential biomarker for RA and is upregulated in FLS in response to TNF stimulation. Intracellular FH cooperates with TNF to establish a feed-forward amplification loop that additively enhances the induction of RA-associated soluble mediators, including GM-CSF, IL-8, and IL-6. At least in vitro, the suppression of intracellular FH attenuates TNF-induced inflammatory gene expression and reduces the production of these mediators.

## Supplementary Information

Additional file 1. List of RT-qPCR primer sequences for human genes.

Additional file 2. Regulation of *F13B* expression by inflammatory cytokines and its effect on *CSF2* expression in FLS*F13B* expression levels in FLS stimulated with TNF or IL-6 plus IL-6Rα for 24 h were detected by RT-qPCR. Data were analyzed using Dunnett’s test. FLS were transfected with F13B IVT mRNA or EGFP IVT mRNA for 24 h. EGFP IVT mRNA was used as a negative control. *CSF2* expression levels in these cells were measured by RT-qPCR. Data were analyzed using Student’s t-test. Abbreviations: TNF, tumor necrosis factor; IL-6, interleukin-6; FLS, fibroblast-like synoviocytes; CFH, complement factor H; EGFP, enhanced green fluorescent protein; F13B, factor XIII B subunit.

Additional file 3. Comparison of plasmid- and IVT mRNA-mediated the expression of FXIII-B and FH in MH7A cells. Immunocytochemistry of MH7A cells 24 h after transfection. Cells were stained with an anti-HA tag antibody or an anti-EGFP antibody, followed by Alexa Fluor 647-conjugated secondary antibody and DAPI. Scale bar = 100 µm.F13B-HA or EGFP plasmids. CFH-HA or EGFP plasmids.F13B-HA IVT mRNA or EGFP IVT mRNA. CFH-HA IVT mRNA or EGFP IVT mRNA. Western blotting analysis of MH7A cells transfected with IVT mRNA for 24 h.F13B-HA IVT mRNA or EGFP IVT mRNA. Cells transfected with CFH-HA IVT mRNA or EGFP IVT mRNA. Total cell lysate. Cytoplasmic fraction. Abbreviations: HA, influenza hemagglutinin; DAPI, 4′,6-diamidino-2-phenylindole; GAPDH, glyceraldehyde-3-phosphate dehydrogenase.

Additional file 4. Plasmid-mediated expression of *F13B* or *CFH* fails to induce inflammatory gene expression. RT-qPCR was performed to detect expression levels of various factorsin MH7A cells that were transfected with *F13B* or *CFH* expression plasmids.*CSF2CXCL8TRAF1CXCL1CXCL2CCL3*. In contrast to IVT mRNA-mediated expression, plasmid-based expression of F13B or CFH did not significantly affect inflammatory gene expression, indicating that intracellular retention is required for their functional effects. Abbreviations: CSF2, colony stimulating factor 2; CXCL8, C-X-C motif chemokine ligand 8; TRAF1, TNF receptor-associated factor 1; CXCL1, C-X-C motif chemokine ligand 1; CXCL2, C-X-C motif chemokine ligand 2; CCL3, C–C motif chemokine ligand 3.

Additional file 5. IVT mRNA-mediated expressions do not associate with alteration in endoplasmic reticulum stress markers. RT-qPCR was performed to assess the expression levels of endoplasmic reticulumstress-related factors in MH7A cells transfected with CFH IVT mRNA or EGFP IVT mRNA, or treated with tunicamycin for 24 h.HSPA5DDIT3.Western blotting analysis of eIF2α phosphorylation in MH7A cells transfected with CFH IVT mRNA or EGFP IVT mRNA, or treated with tunicamycin for 4 h. Abbreviations: HSPA5, heat shock protein family Amember 5; DDIT3, DNA damage inducible transcript 3; eIF2α, eukaryotic translation initiation factor 2A.

Additional file 6. Gene ontology analysis of RNA-seq data shown in Fig. 3A. RNA was collected from FLS transfected with CFH IVT mRNA or EGFP IVT mRNA. RNA-seq analysis was performed to examine FH-induced gene expression in primary cultured FLS. This table shows the list of functional annotations for upregulated genes. The ontology analysis was performed using DAVID Bioinformatics Resources v2025, accessed on July 7, 2026.

Additional file 7. NOD2 is detected in an FH-containing protein complex. MH7A was transfected with CFH IVT mRNA. Cell lysates were immunoprecipitated with either an anti-HA-tag antibody or control IgG, followed by capture with Protein G beads. The eluted samples were analyzed using western blotting. Input and IP panels were exposed for different durations. The white arrowhead indicates the band corresponding to endogenous NOD2. Abbreviations: IP, immunoprecipitation; FH, factor H; NOD2, nucleotide‐binding oligomerization domain 2.

Additional file 8. FH-induced *TRAF1* expression is associated with increased MH7A cell proliferation.MH7A cells were transfected with CFH IVT mRNA, EGFP IVT mRNA, or mock IVT mRNA. Cells were additionally treated with a NOD2 inhibitor. RT-qPCR showed that FH-induced TRAF1 expression was suppressed by GSK717.Cell viability assay using calcein-AM at 24 h after transfection. Cell numbers in CFH IVT mRNA-transfected cells were higher than in controls.MH7A cells were transfected with 50 nM siRNA against CFHor control siRNA. Cells were harvested 96 h after transfection. RT-qPCR analysis showed decreased TRAF1 expression following CFH knockdown. Cell viability assay using calcein-AM. Cell numbers in siCFH-treated cells were lower than in siControl.

Additional file 9. Anti-FH antibody does not suppress TNF-induced cytokine gene expression.MH7A cells were transfected with either CFH IVT mRNA or EGFP IVT mRNA. Cells were also treated with anti-FH antibody, control IgG, and/or TNF. IVT mRNA was transfected for 10 h, after which neutralization was performed, followed by the addition of TNF for 12 h. The expression levels of the following cytokines were measured by RT-qPCR:*CSF2*,*CXCL8*, and*IL6*. Abbreviations: FH, factor H; IgG, immunoglobulin G.

Additional file 10. Relative expression levels of CFH family genes in FLS. FLS samples were obtained from three individual subjects. Total RNA from primary cultured FLS was analyzed using RNA-seq. Expression values were normalized using the TPM method. Abbreviations: CFHR, complement factor H related; TPM, transcripts per million.

Additional file 11.

## Data Availability

The datasets analyzed in this study are available from the corresponding author upon reasonable request. Certain data cannot be shared because these data include information that may compromise participant privacy. The RNA-seq raw data have been deposited in the DDBJ Sequence Read Archive (DRA) under BioProject ID PRJDB39751, and in the NCBI Sequence Read Archive with accession number PRJNA915654.

## Abbreviations

aHUS: Atypical hemolytic uremic syndrome
CCL3: C-C motif chemokine ligand 3
CDH11: Cadherin-11
CFH: Complement factor H (for gene name)
CFHR: Complement factor H-related (for gene name)
Co-IP: Co-immunoprecipitation
csDMARDs: Conventional synthetic disease-modifying antirheumatic drugs
CXCL8: C-X-C motif chemokine ligand 8
DDIT3: DNA damage inducible transcript 3
EGFP: Enhanced green fluorescent protein
ELISA: Enzyme-linked immunosorbent assay
eIF2α: Eukaryotic translation initiation factor 2A
ER: Endoplasmic reticulum
FFPE: Formalin-fixed paraffin-embedded
FH: Factor H
FHR: Factor H-related
FLS: Fibroblast-like synoviocytes
FXIII-B: Factor XIII B
F13B: Factor XIII B (for gene name)
GM-CSF: Granulocyte-macrophage colony-stimulating factor
GTF: Gene transfer format
HSPA5: Heat shock protein family A (Hsp70) member 5
IL: Interleukin
IVT mRNA: In vitro transcribed messenger RNA
MDP: Muramyl dipeptide
NOD2: Nucleotide-binding oligomerization domain 2
OA: Osteoarthritis
RA: Rheumatoid arthritis
RCA: Regulators of complement activation
RIPK2: Receptor-interacting serine/threonine-protein kinase 2
siRNA: Small interfering RNA
TNF: Tumor necrosis factor
TPM: Transcripts per million
TRAF1: TNF receptor associated factor 1 (TRAF1)
