# Supplementary material for: TNF-driven intracellular complement factor H–NF-κB feed-forward loop amplifies inflammatory mediator production in rheumatoid arthritis synovial fibroblasts
Source: Inflamm Regen. 2026 Aug 4;46:40. doi: 10.1186/s41232-026-00437-1 (PMC13721194; doi:10.1186/s41232-026-00437-1)

Additional Files

TNF-driven intracellular complement factor H–NF-κB feed-forward loop amplifies inflammatory mediator production in rheumatoid arthritis synovial fibroblasts

Kobayashi H and Mokuda S, et al.

- Additional File 1, 2, 3, 4, 5, 6, 7, 8, 9 and 10
- Uncropped blotting data for WB

Additional File 1

Gene symbol	Sequence (5'-3') for forward	Sequence (5'-3') for reverse
CCL3	CTCTGCAACCAGTTCTC	AATTCTGTGGAATCTGCC
CFH	TTCCGTTCAGTGCTACCACTTTG	CATTGAGGAGTTCAGGAGGTG
CSF2	GAGCATGTGAATGCCATCCAG	TGGAGGTCAAACATTTCTGAGAT
CXCL1	TGCTGAACAGTGACAAATC	CTTCTGTTCCTATAAGGGC
CXCL2	GCAGAAAGCTTGTCTCAACCC	CTCCTTCAGGAACAGCCACCAA
CXCL8	GTTTTTGAAGAGGGCTGAG	TTTGCTTGAAGTTTCACTGG
DDIT3	GTATGAGGACCTGCAAGAGGT	TTGTGACCTCTGCTGGTTCTG
F13B	AAGCGGCTACCTTCTCCATGG	CCAATATCCCGTCTGCAACAG
GAPDH	CTTTTGCGTCGCCAG	TTGATGGCAACAATATCCAC
HSPA5	TGTCCAGGCTGGTGTGCTCT	TTGGTAGGCACCACTGTGTTC
IL6	GCAGAAAAAGGCAAGAATC	CTACATTTGCGGAAGAGC
TRAF1	GATGGCACTTTCCTGTGGAAG	ACAGCCGCAGGCACAACTTGT

Additional File 1. List of RT-qPCR primer sequences for human genes

Abbreviations: CCL3, C-C motif chemokine ligand 3; CFH, complement factor H; CSF2, colony stimulating factor 2; CXCL1, C-X-C motif chemokine ligand 1; CXCL2, C-X-C motif chemokine ligand 2; CXCL8, C-X-C motif chemokine ligand 8; DDIT3, DNA damage inducible transcript 3; F13B, factor XIII B; GAPDH, glyceraldehyde-3-phosphate dehydrogenase; HSPA5, heat shock protein family A (Hsp70) member 5; IL6, interleukin 6; TRAF1, TNF receptor-associated factor 1.

Additional File 2

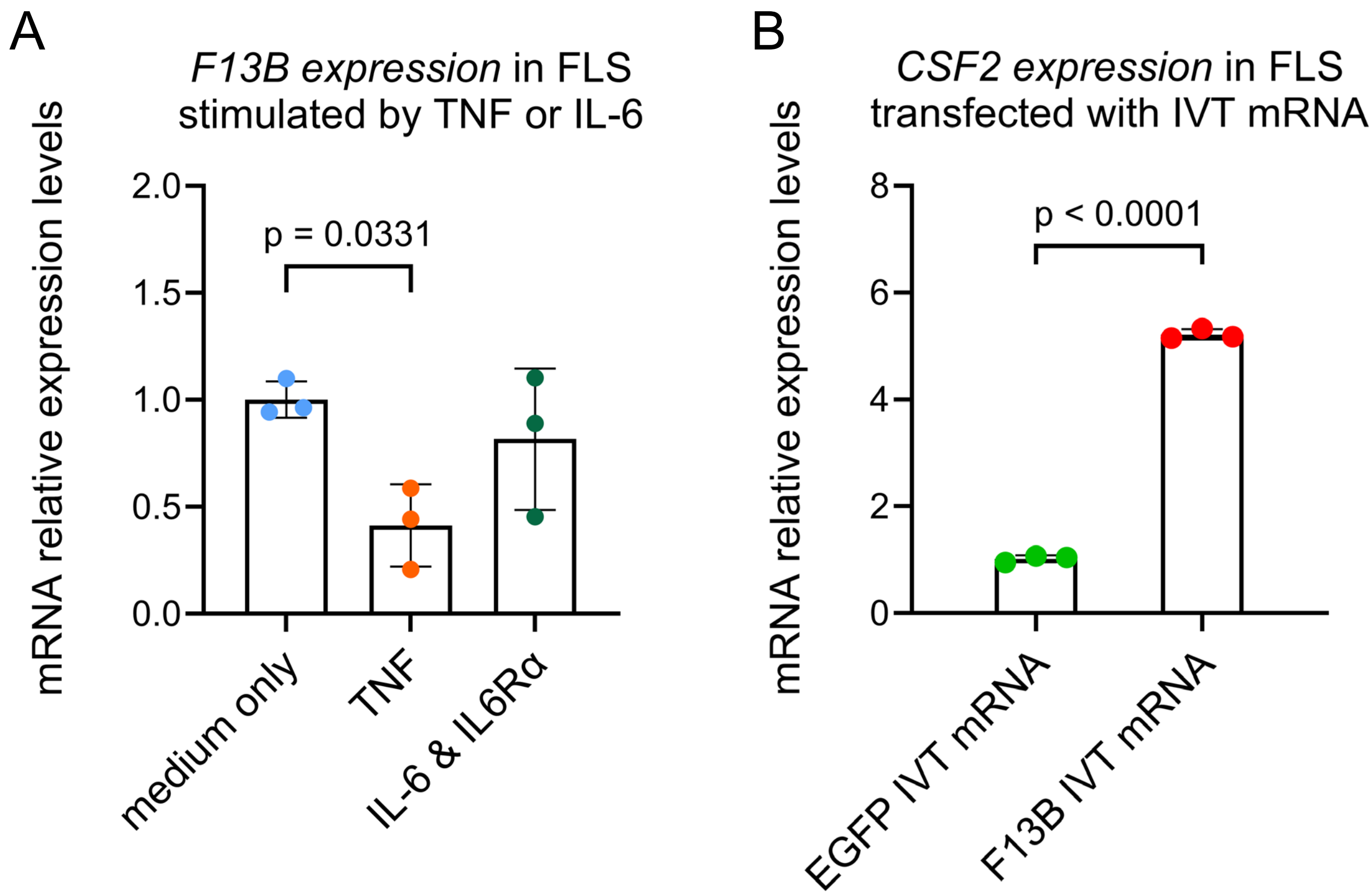

Additional File 2. Regulation of *F13B* expression by inflammatory cytokines and its effect on *CSF2* expression in FLS

(A) *F13B* expression levels in FLS stimulated with TNF or IL-6 plus IL-6Rα for 24 hours were detected by RT-qPCR (n = 3, each from an individual subject). Data were analyzed using Dunnett's test. (B) FLS were transfected with F13B IVT mRNA or EGFP IVT mRNA for 24 hours. EGFP IVT mRNA was used as a negative control. *CSF2* expression levels in these cells were measured by RT-qPCR (n = 3). Data were analyzed using Student's t-test. Abbreviations: TNF, tumor necrosis factor; IL-6, interleukin-6; FLS, fibroblast-like synoviocytes; CFH, complement factor H; EGFP, enhanced green fluorescent protein; F13B, factor XIII B.

Additional File 3

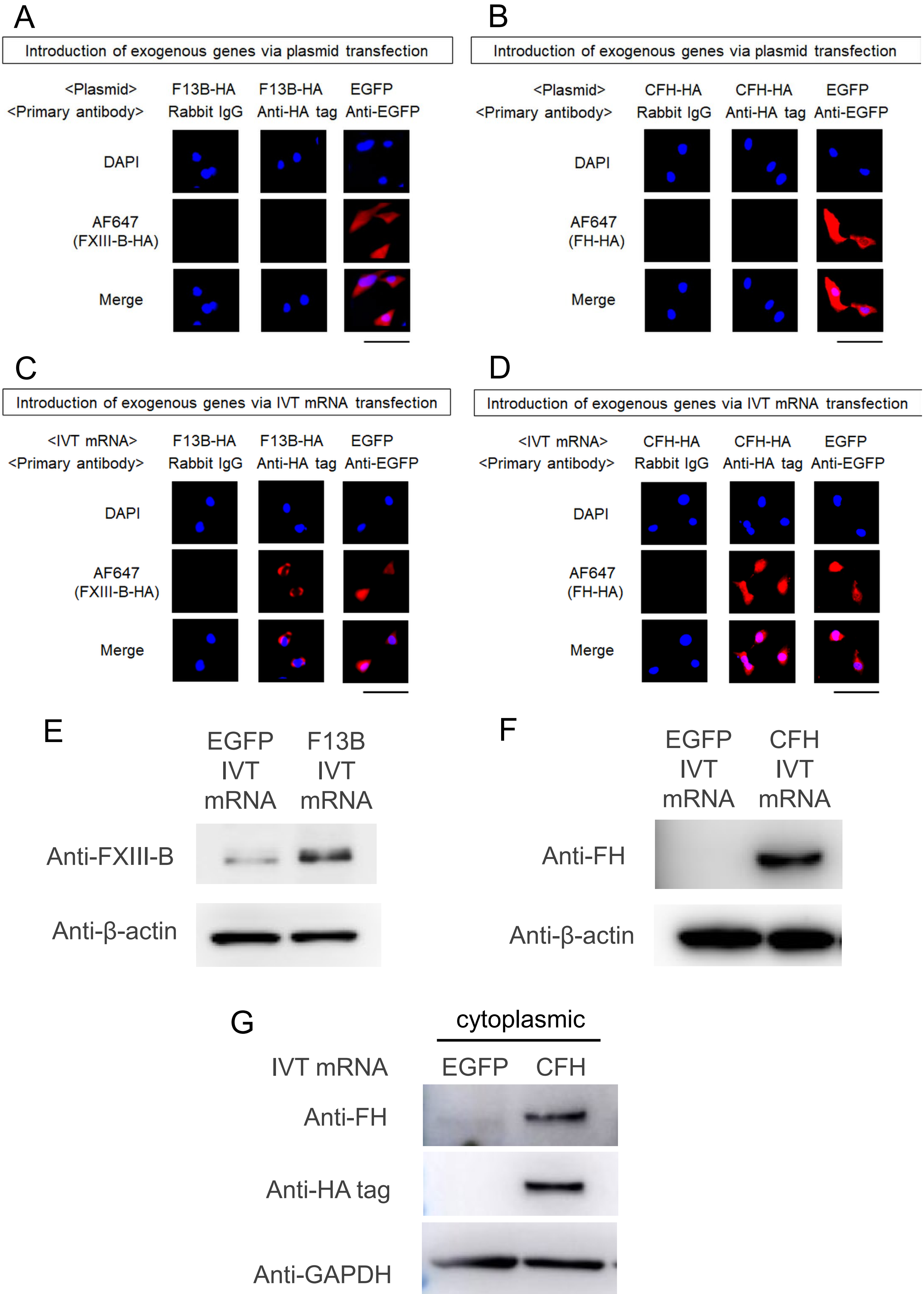

Additional File 3. Comparison of plasmid- and IVT mRNA-mediated expression of FXIII-B and FH in MH7A cells

(Continue to next page)

Additional File 3. Comparison of plasmid- and IVT mRNA-mediated expression of FXIII-B and FH in MH7A cells

(A–D) Immunocytochemistry of MH7A cells 24 hours after transfection. Cells were stained with an anti-HA tag antibody or an anti-EGFP antibody, followed by Alexa Fluor 647-conjugated secondary antibody and DAPI. Scale bar = 100 μm. (A) F13B-HA or EGFP plasmids. (B) CFH-HA or EGFP plasmids. (C) F13B-HA IVT mRNA or EGFP IVT mRNA. (D) CFH-HA IVT mRNA or EGFP IVT mRNA. (E, F, G) Western blotting analysis of MH7A cells transfected with IVT mRNA for 24 hours. (E) F13B-HA IVT mRNA or EGFP IVT mRNA. (F, G) Cells transfected with CFH-HA IVT mRNA or EGFP IVT mRNA. (F) Total cell lysate. (G) Cytoplasmic fraction. Abbreviations: HA, influenza hemagglutinin; DAPI, 4′,6-diamidino-2-phenylindole; GAPDH, glyceraldehyde-3-phosphate dehydrogenase.

Additional File 4

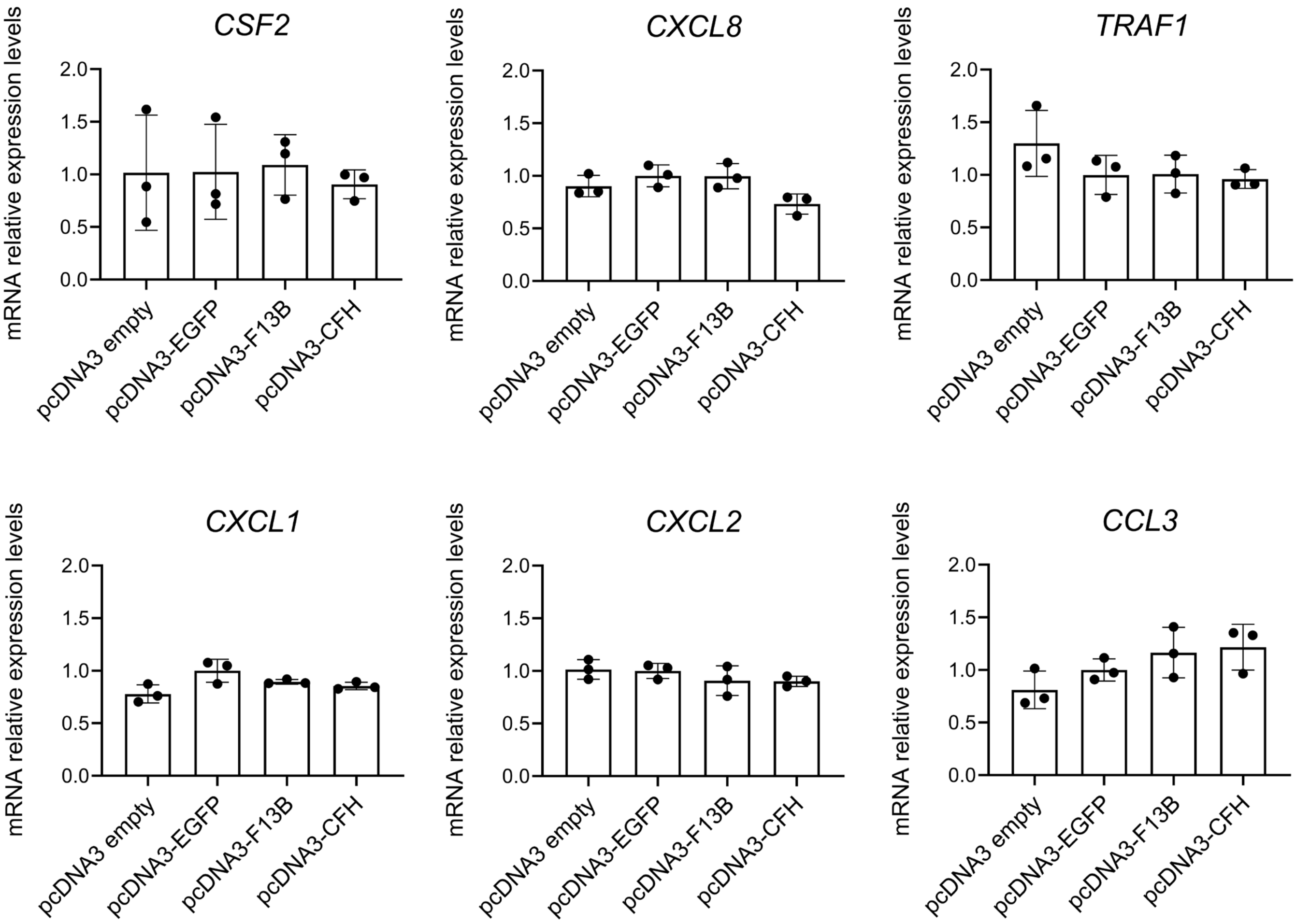

Additional File 4. Plasmid-mediated expression of *F13B* or *CFH* fails to induce inflammatory gene expression

RT-qPCR was performed to detect the expression levels of various factors (e.g., *CSF2, CXCL8, TRAF1, CXCL1, CXCL2*, and *CCL3*) in MH7A cells that were transfected with *F13B* or *CFH* expression plasmids (n = 3). (A) *CSF2* (B) *CXCL8* (C) *TRAF1* (D) *CXCL1* (E) *CXCL2* (F) *CCL3*. In contrast to IVT mRNA–mediated expression, plasmid-based expression of *F13B* or *CFH* did not significantly affect inflammatory gene expression, indicating that intracellular retention is required for their functional effects. Abbreviations: CSF2, colony stimulating factor 2; CXCL8, C-X-C motif chemokine ligand 8; TRAF1, TNF receptor-associated factor 1; CXCL1, C-X-C motif chemokine ligand 1; CXCL2, C-X-C motif chemokine ligand 2; CCL3, C-C motif chemokine ligand 3.

Additional File 5

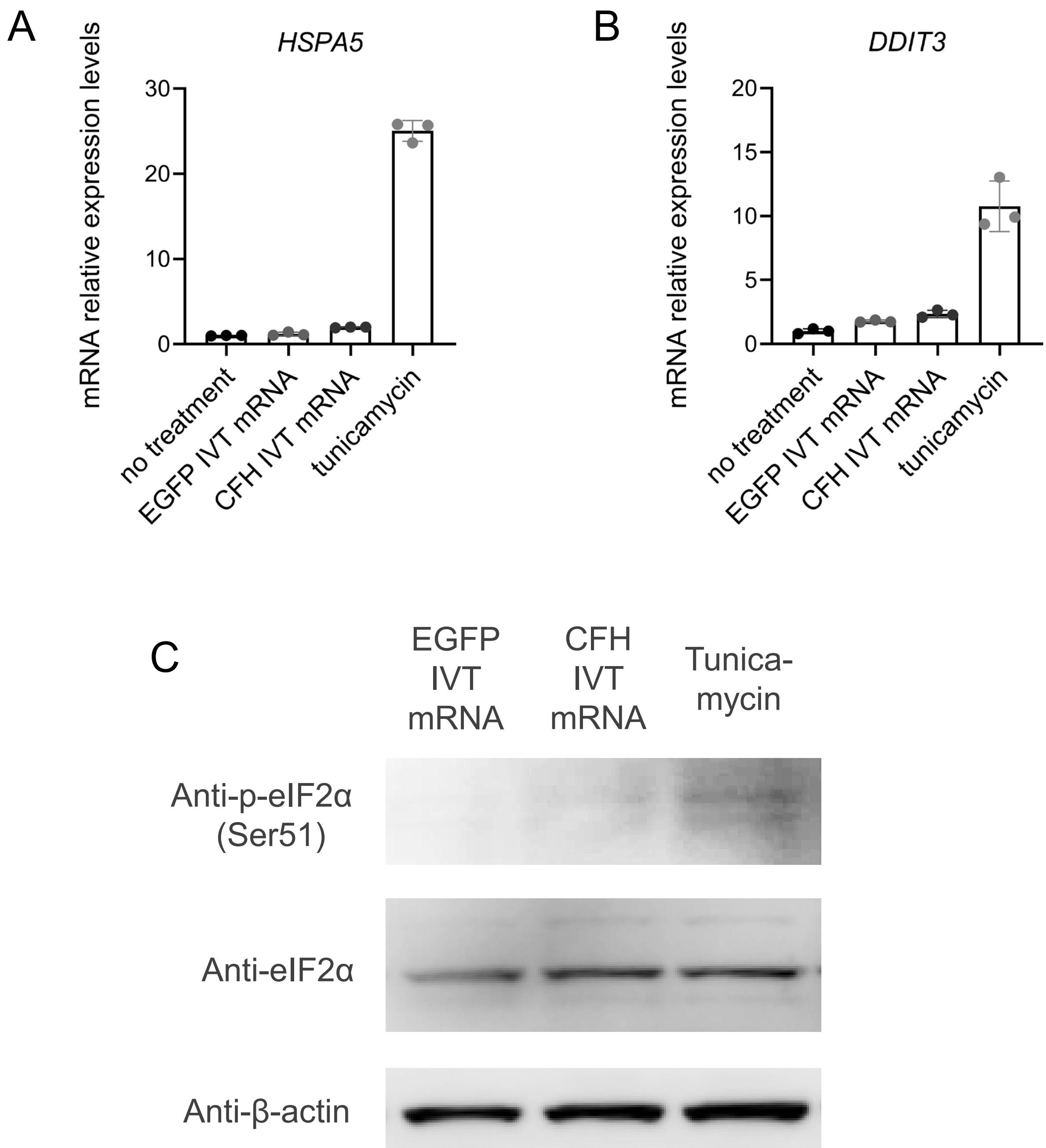

Additional File 5. IVT mRNA-mediated expressions do not associate with alteration in endoplasmic reticulum stress markers

(A, B) RT-qPCR was performed to assess the expression levels of endoplasmic reticulum (ER) stress-related factors in MH7A cells transfected with CFH IVT mRNA or EGFP IVT mRNA, or treated with tunicamycin for 24 hours (n = 3). (A) HSPA5 (B) DDIT3. (C) Western blotting analysis of eIF2α phosphorylation in MH7A cells transfected with CFH IVT mRNA or EGFP IVT mRNA, or treated with tunicamycin for 4 hours.
Abbreviations: HSPA5, heat shock protein family A (Hsp70) member 5; DDIT3, DNA damage inducible transcript 3; eIF2α, eukaryotic translation initiation factor 2A.

Additional File 6

Functional Annotation Chart in DAVID analysis (Pathway Analysis)

Term	Count	P-Value
Cytokine-cytokine receptor interaction	14	6.8E-11
Viral protein interaction with cytokine and cytokine receptor	9	1.7E-08
TNF signaling pathway	9	4.7E-08
Chemokine signaling pathway	10	7.2E-08
IL-17 signaling pathway	8	1.5E-07
Rheumatoid arthritis (＊)	8	1.5E-07
NF-kappa B signaling pathway	6	1.9E-04
Legionellosis	5	2.8E-04
Lipid and atherosclerosis	7	4.1E-04
Epithelial cell signaling in Helicobacter pylori infection	5	5.7E-04

(＊) *CXCL8, CSF2, CXCL1, CXCL2* were included.

Additional File 6. Gene ontology analysis of RNA-seq data shown in Figure 3A

RNA was collected from FLS transfected with CFH IVT mRNA or EGFP IVT mRNA (negative control). RNA-seq analysis was performed to examine FH-induced gene expression in primary cultured FLS (n = 4, each from an individual subject). This table shows the list of functional annotations for upregulated genes. The ontology analysis was performed using DAVID Bioinformatics Resources v2025 (https://david.ncifcrf.gov/), accessed on July 7, 2026.

Additional File 7

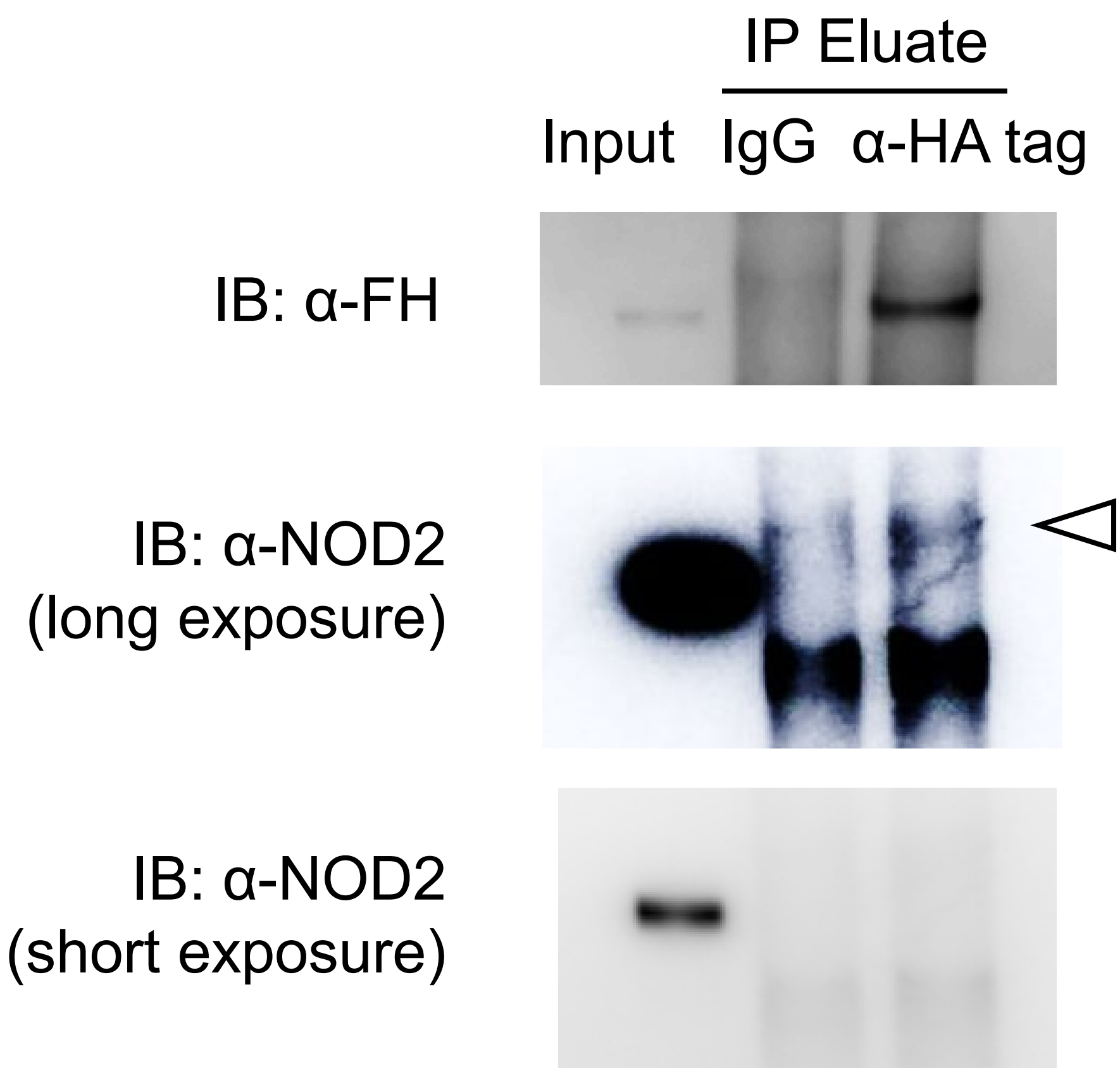

Additional File 7. NOD2 is detected in an FH-containing protein complex

MH7A was transfected with CFH IVT mRNA. Cell lysates were immunoprecipitated with either an anti-HA-tag antibody or control IgG, followed by capture with Protein G beads. The eluted samples were analyzed using western blotting. Input and IP panels were exposed for different durations. The white arrowhead indicates the band corresponding to endogenous NOD2.

Abbreviations: IP, immunoprecipitation; FH, factor H; NOD2, nucleotide-binding oligomerization domain 2.

Additional File 8

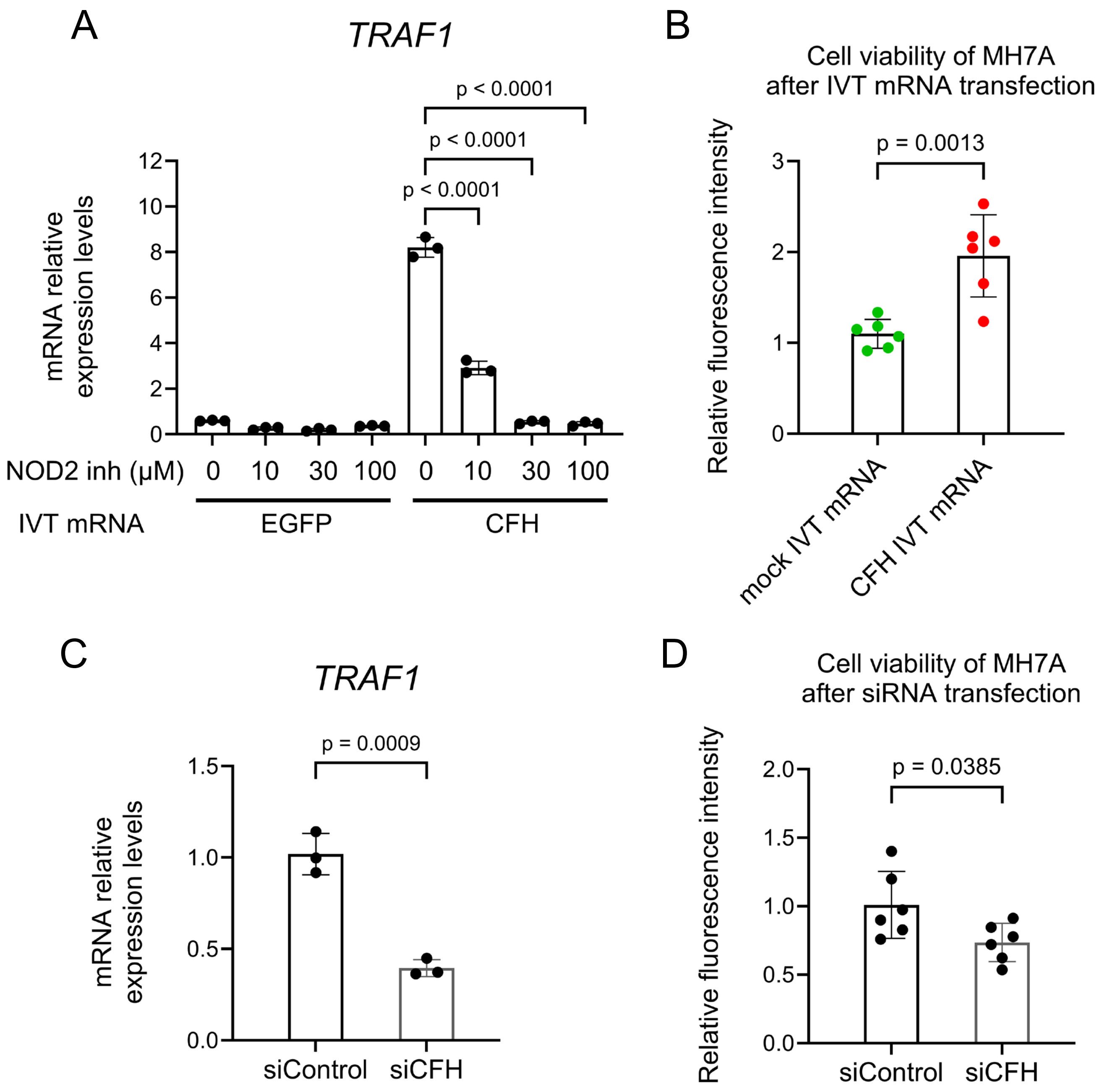

Additional File 8. FH-induced *TRAF1* expression is associated with increased MH7A cell proliferation

(A, B) MH7A cells were transfected with CFH IVT mRNA, EGFP IVT mRNA, or mock IVT mRNA. (A) Cells were additionally treated with a NOD2 inhibitor (GSK717, a competitive antagonist). RT-qPCR showed that FH-induced *TRAF1* expression was suppressed by GSK717 (n = 3, Dunnett's test). (B) Cell viability assay using calcein-AM at 24 hours after transfection. Cell numbers in CFH IVT mRNA-transfected cells were higher than in controls (n = 6, Student's t-test). (C, D) MH7A cells were transfected with 50 nM siRNA against *CFH* (siCFH) or control siRNA (siControl). Cells were harvested 96 hours after transfection. (C) RT-qPCR analysis showed decreased *TRAF1* expression following *CFH* knockdown (n = 3, Student's t-test). (D) Cell viability assay using calcein-AM. Cell numbers in siCFH-treated cells were lower than in siControl (n = 6, Student's t-test).

Additional File 9

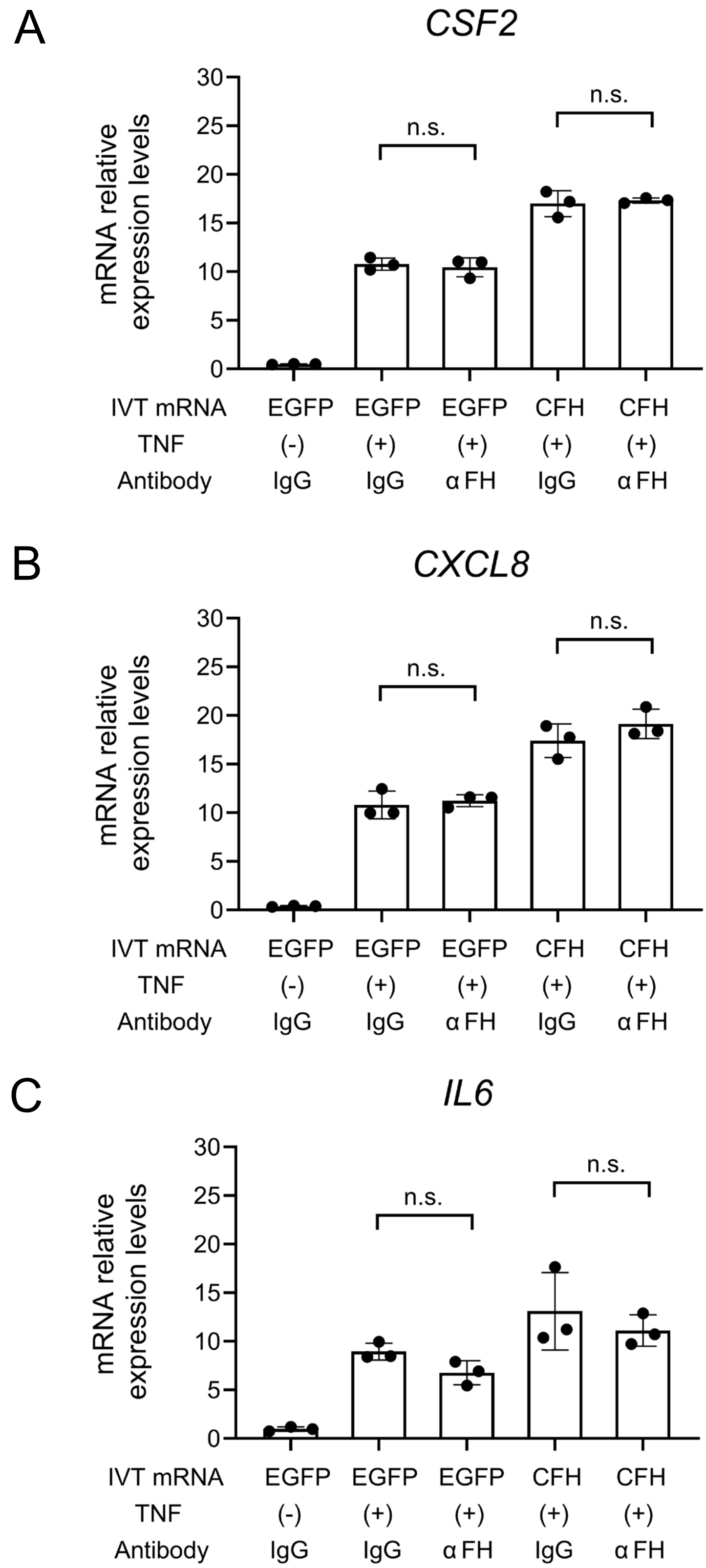

Additional File 9. The anti-FH antibody does not suppress TNF-induced cytokine gene expression

(A–C) MH7A cells were transfected with either CFH IVT mRNA or EGFP IVT mRNA. Cells were also treated with anti-FH antibody, control IgG, and/or TNF. IVT mRNA was transfected for 10 hours, after which neutralization was performed, followed by the addition of TNF for 12 hours. The expression levels of the following cytokines were measured by RT-qPCR (n = 3): (A) *CSF2*, (B) *CXCL8*, and (C) *IL6*. Abbreviations: FH, factor H; IgG, immunoglobulin G.

Additional File 10

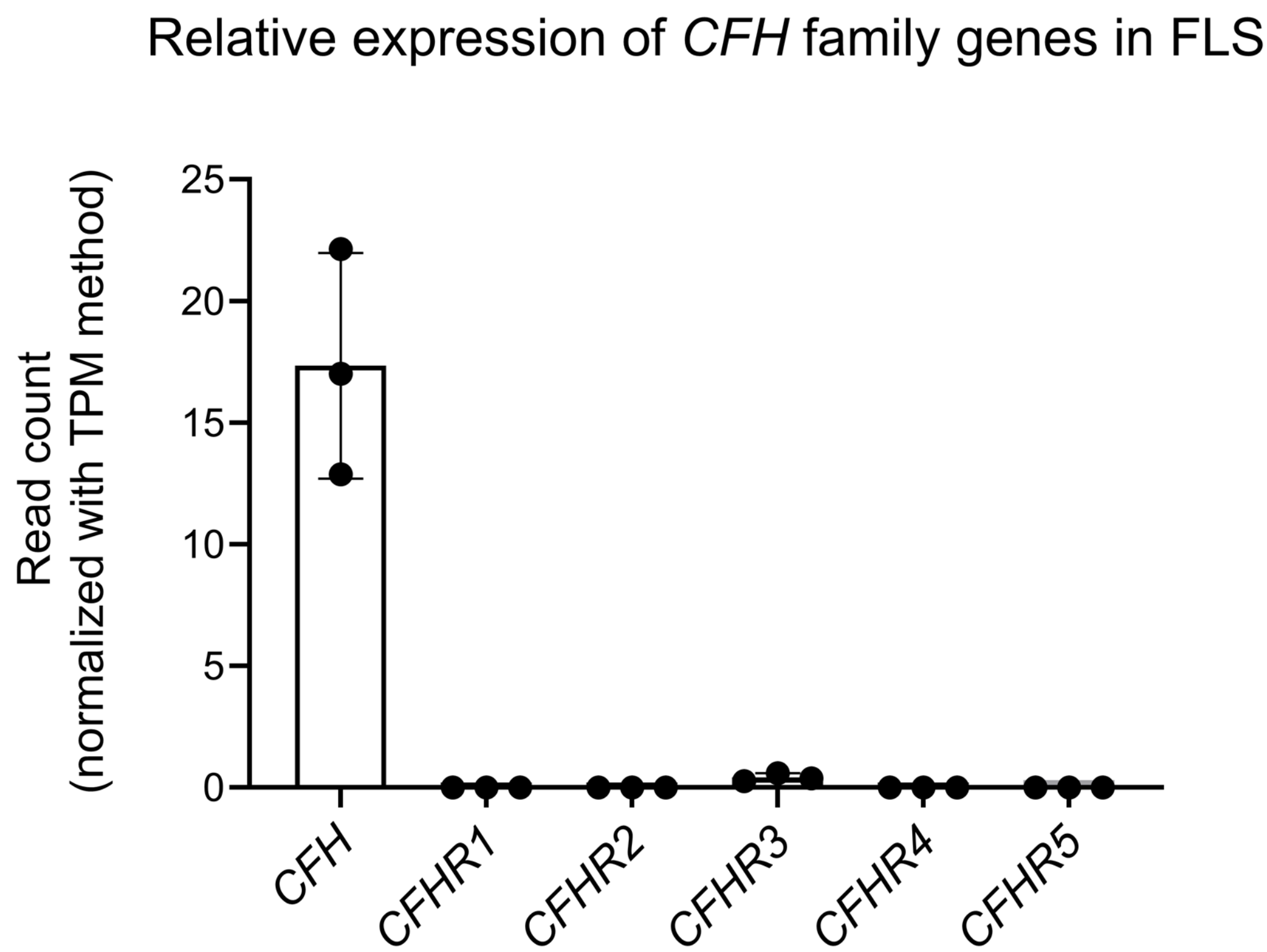

Additional File 10. Relative expression levels of CFH family genes in FLS

FLS samples were obtained from three individual subjects. Total RNA from primary cultured FLS was analyzed using RNA-seq. Expression values were normalized using the TPM method.

Abbreviations: CFHR, complement factor H related; TPM, transcripts per million

Uncropped images for WB

Figure 1F

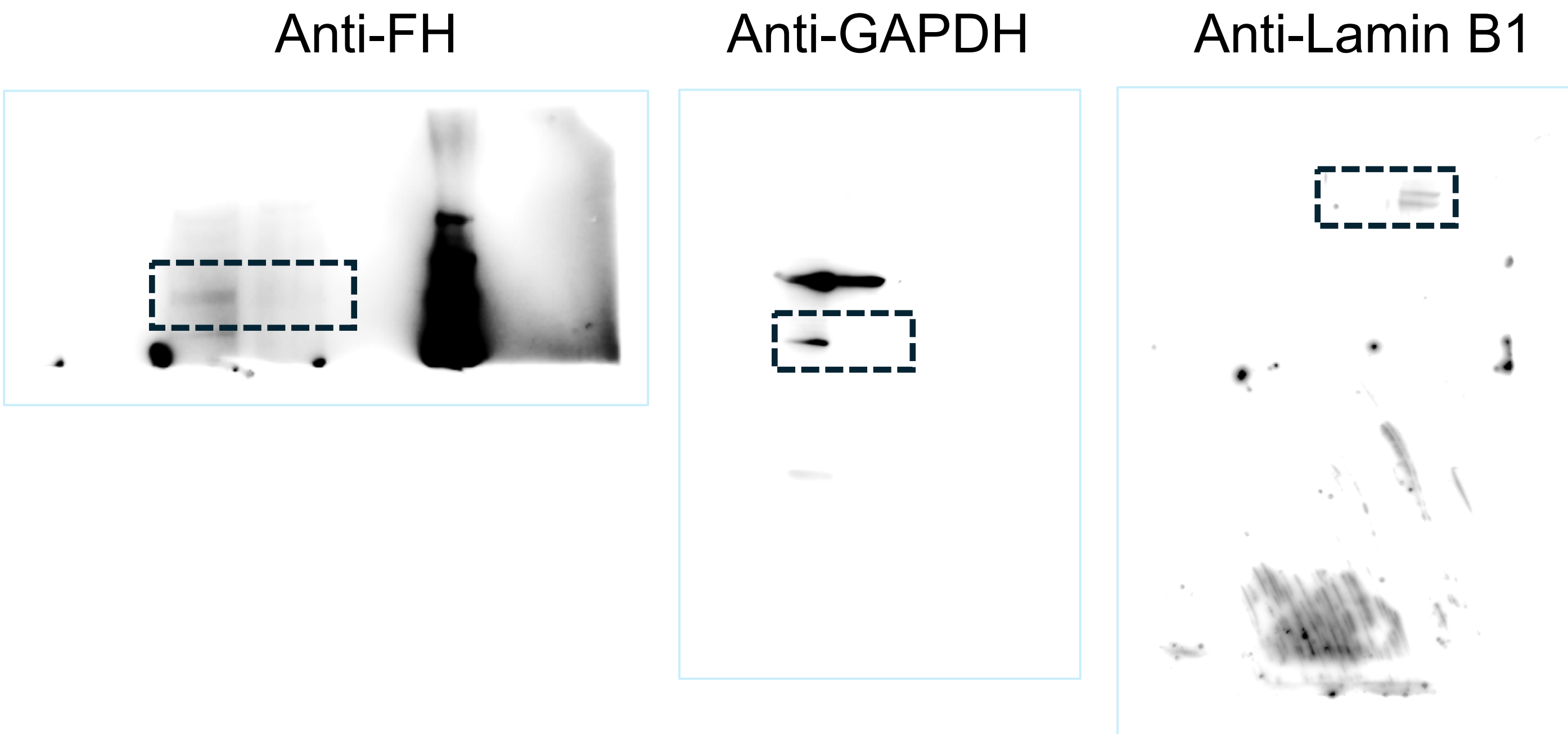

Figure 2F

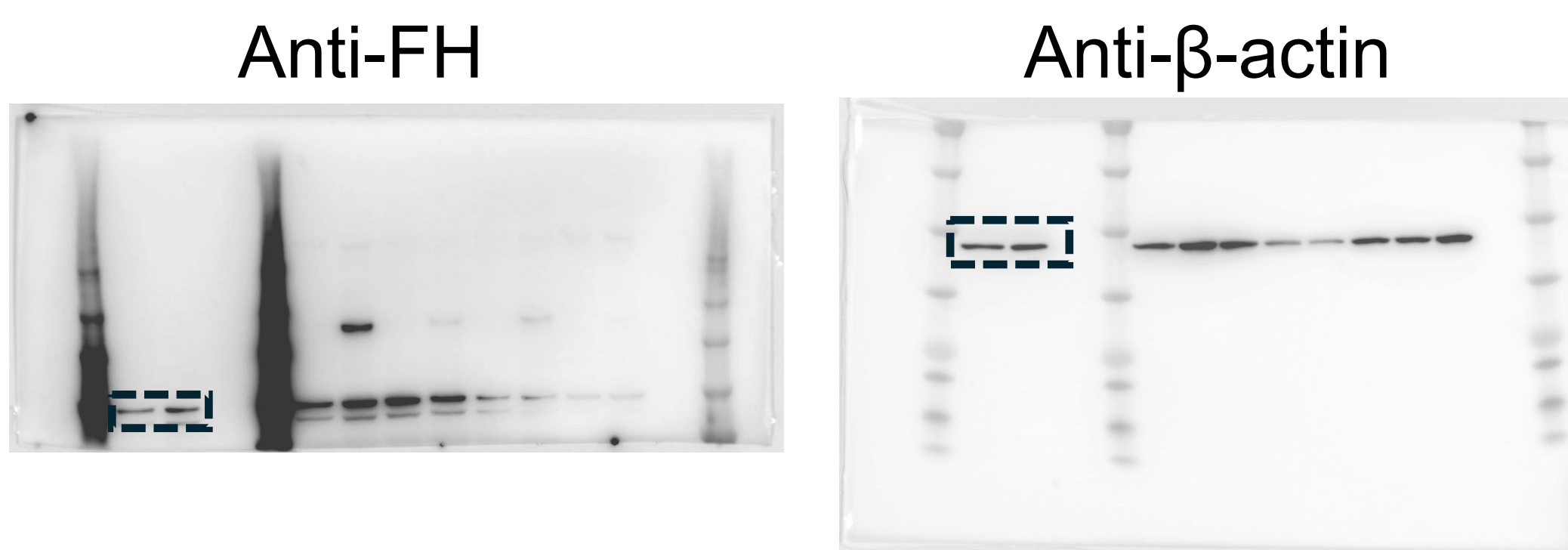

Figure 4D

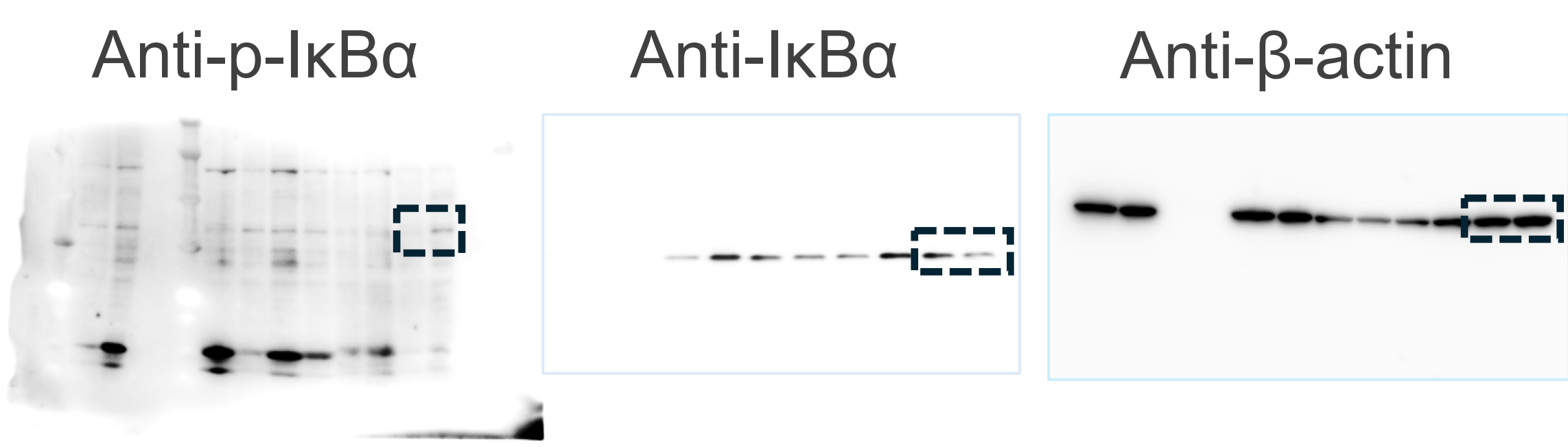

Figure 4E

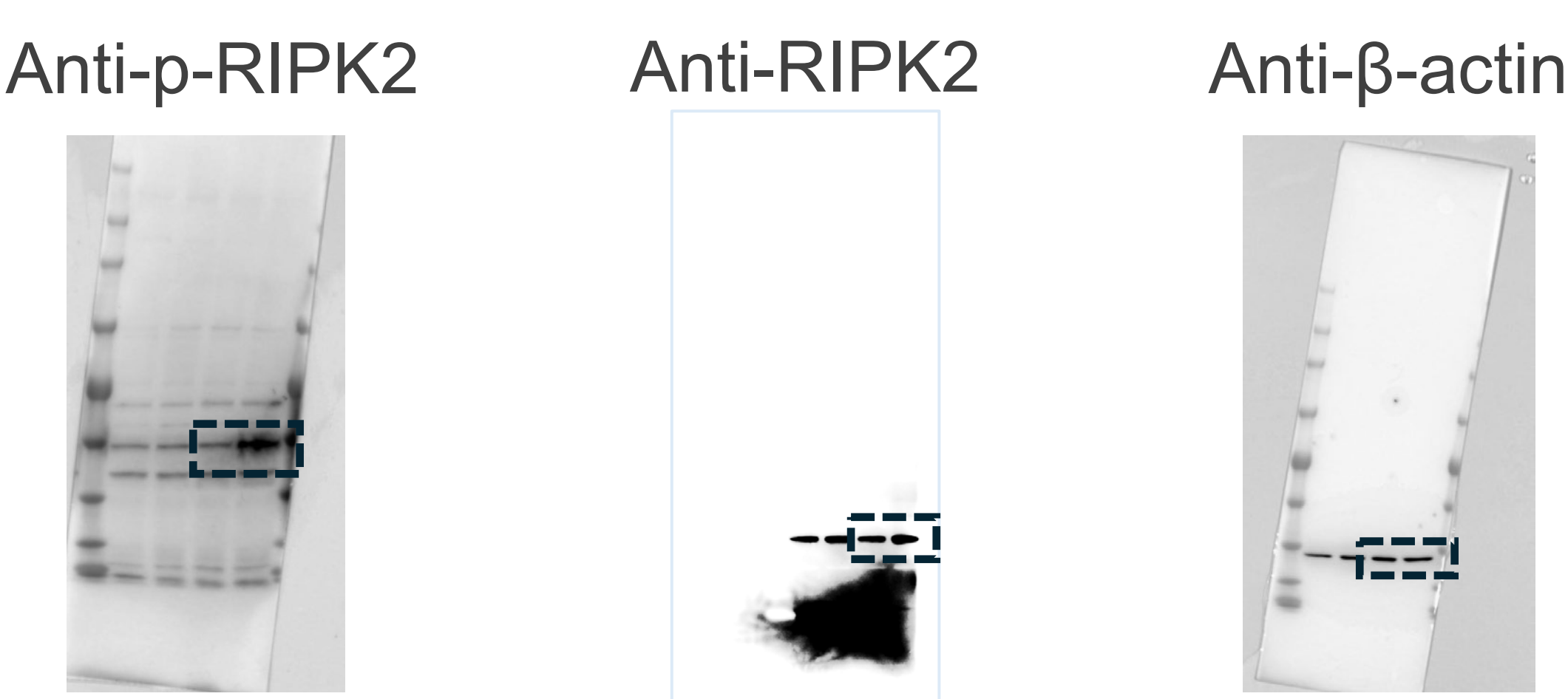

Additional File 3E

Anti-FXIII-B

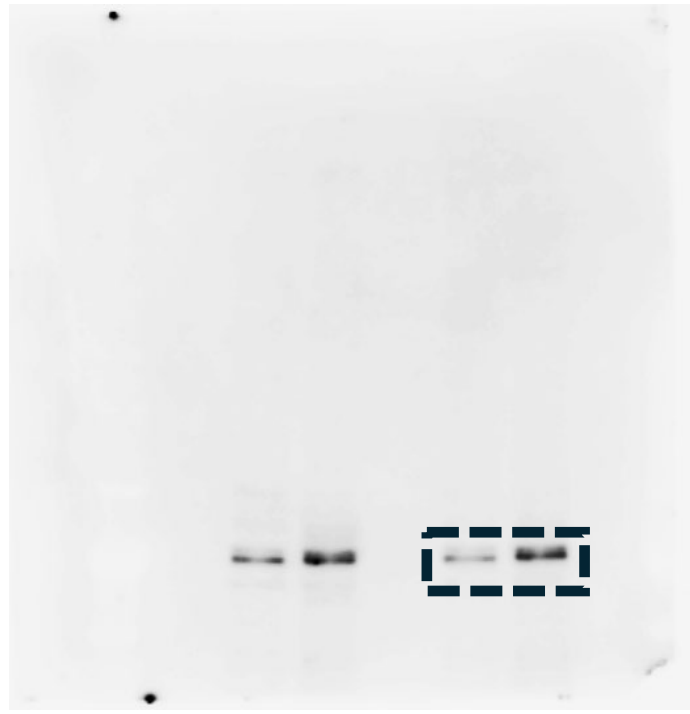

Anti-β-actin

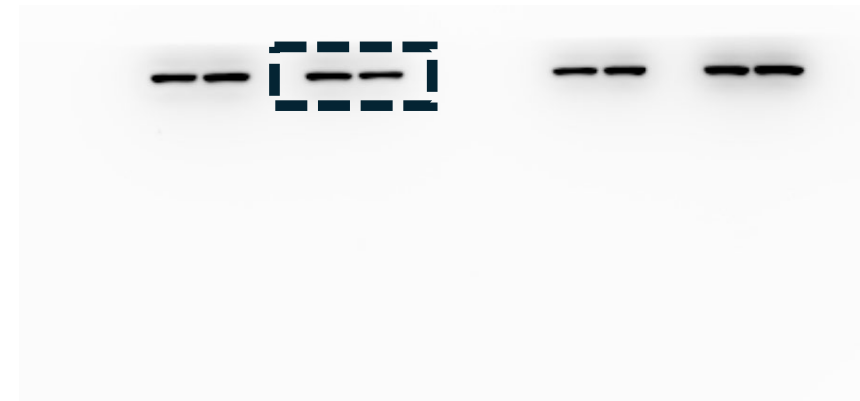

Additional File 3F

Anti-FH

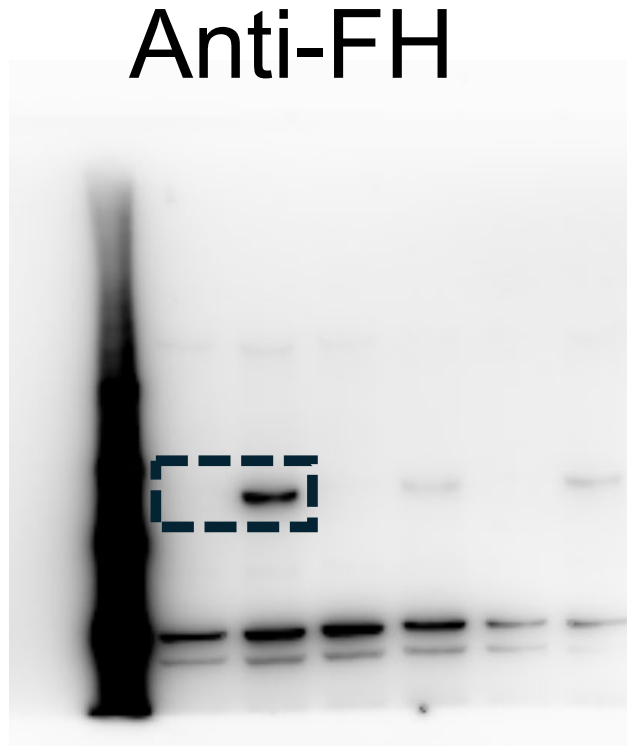

Anti-β-actin

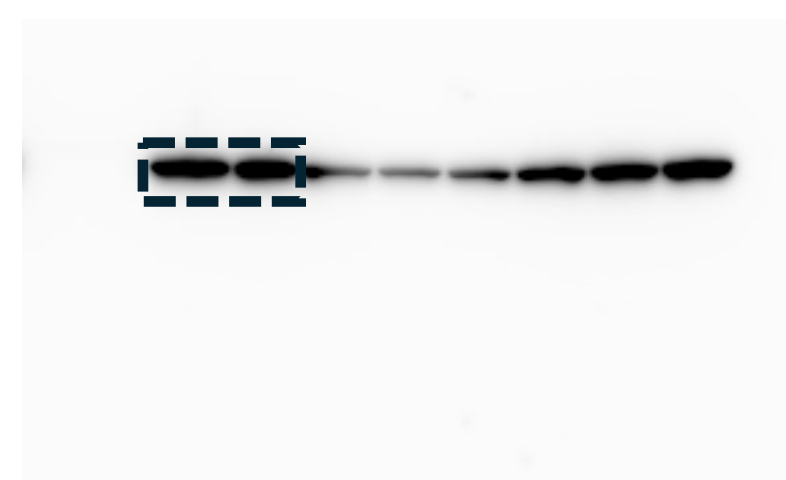

Additional File 3G

Anti-FH

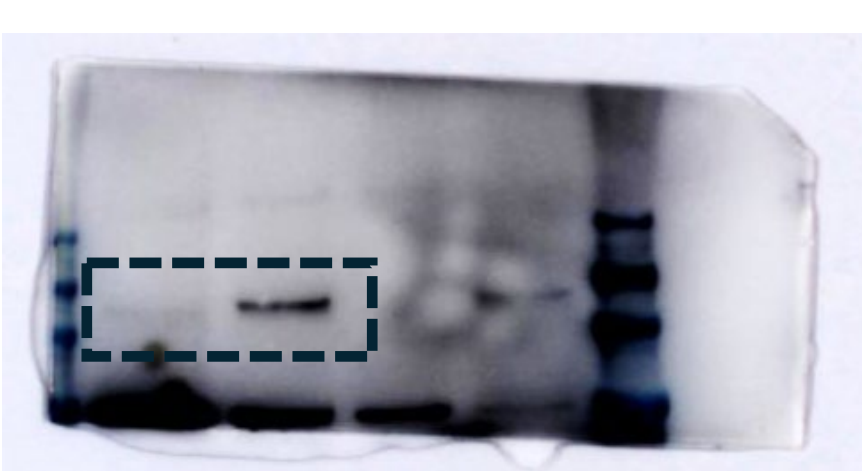

Anti-HA tag

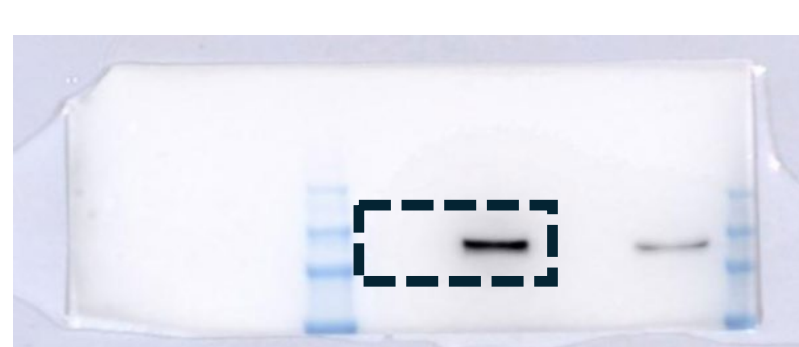

Anti-GAPDH

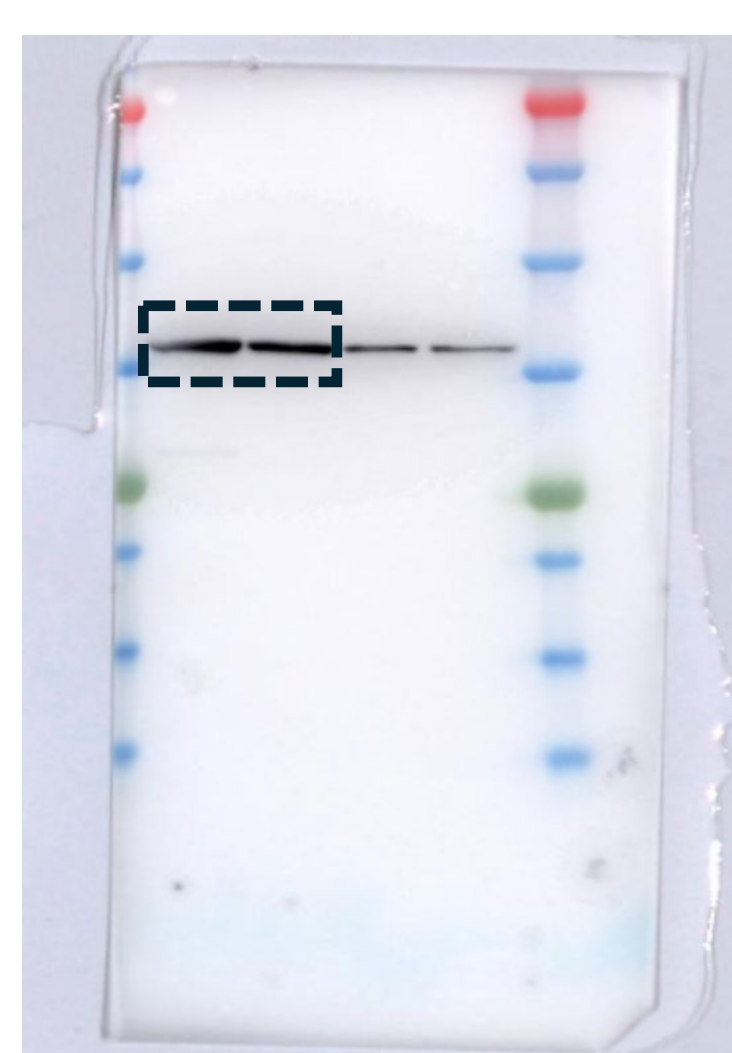

Additional
File 5C

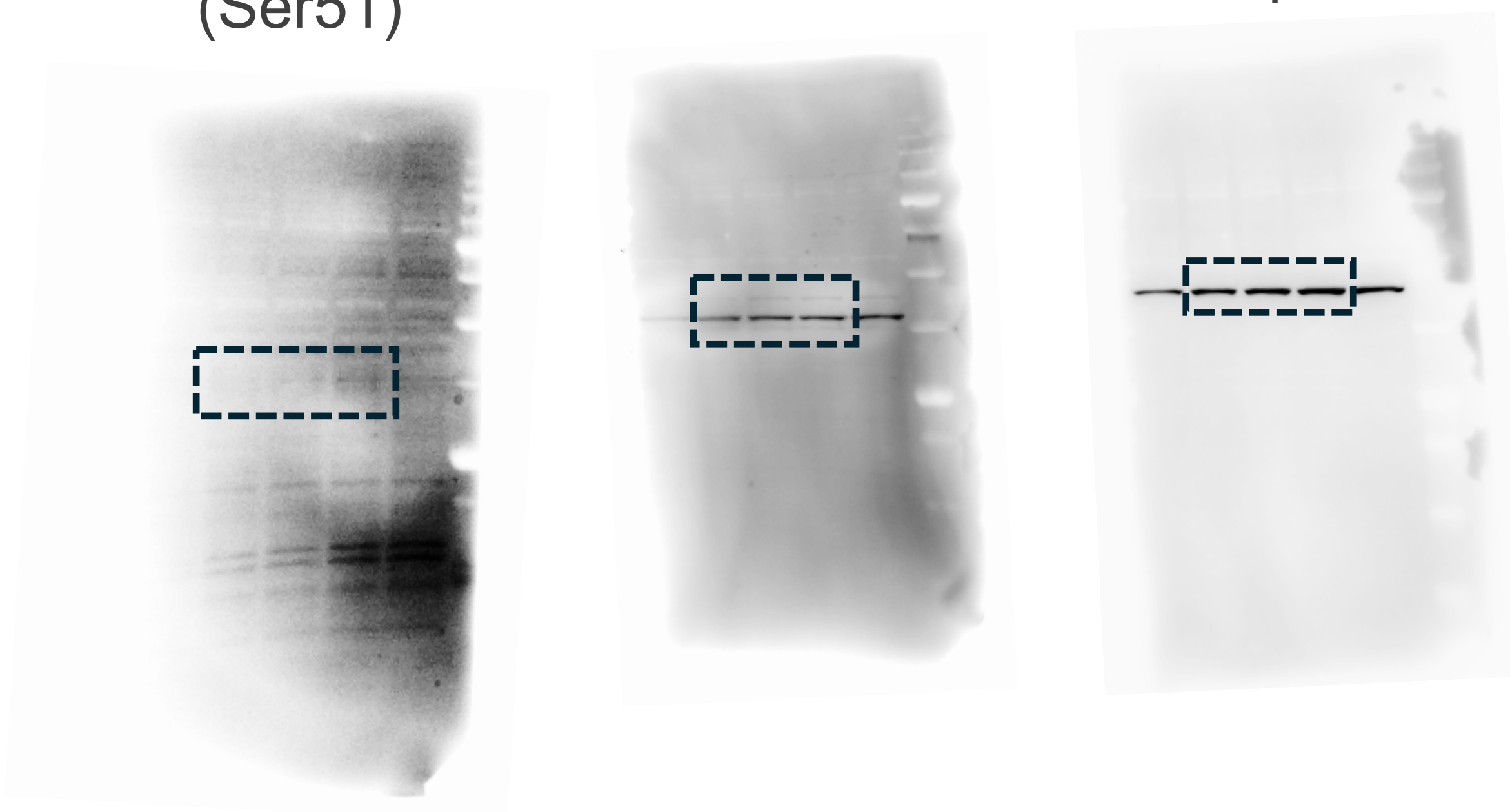

Additional
File 7

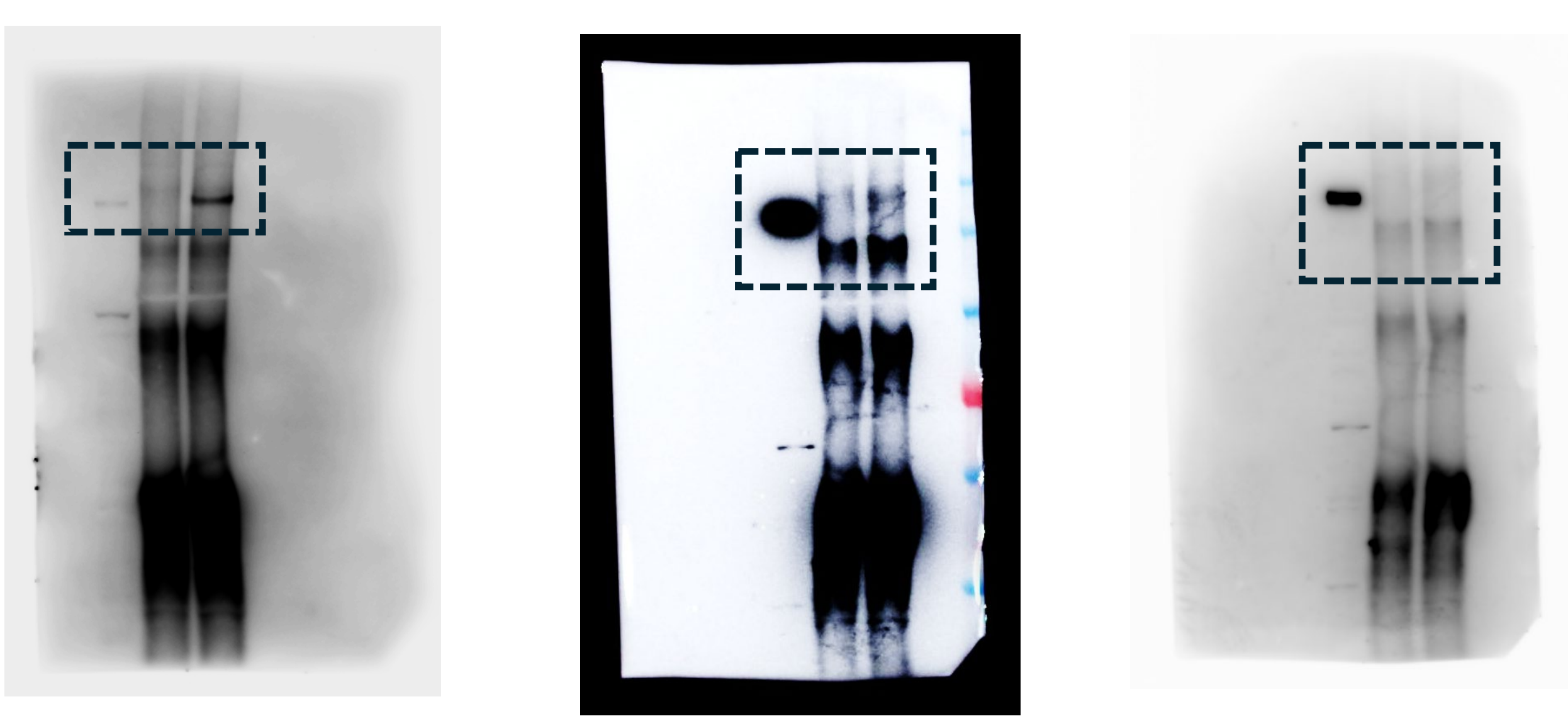

Supplement: Supplementary file 1 [file 41232_2026_437_MOESM11_ESM.pdf]
