# Supplementary material for: TNF-driven intracellular complement factor H–NF-κB feed-forward loop amplifies inflammatory mediator production in rheumatoid arthritis synovial fibroblasts
Source: Inflamm Regen. 2026 Aug 4;46:40. doi: 10.1186/s41232-026-00437-1 (PMC13721194; doi:10.1186/s41232-026-00437-1)
Supplement: Supplementary file 2 [file 41232_2026_437_MOESM10_ESM.docx]

**Additional File 10. Relative expression levels of CFH family genes in FLS**

FLS samples were obtained from three individual subjects. Total RNA from primary cultured FLS was analyzed using RNA-seq. Expression values were normalized using the TPM method.

Abbreviations: CFHR, complement factor H related; TPM, transcripts per million
