# Supplementary material for: TNF-driven intracellular complement factor H–NF-κB feed-forward loop amplifies inflammatory mediator production in rheumatoid arthritis synovial fibroblasts
Source: Inflamm Regen. 2026 Aug 4;46:40. doi: 10.1186/s41232-026-00437-1 (PMC13721194; doi:10.1186/s41232-026-00437-1)
Supplement: Supplementary file 4 [file 41232_2026_437_MOESM2_ESM.docx]

**Additional File 2. Regulation of *F13B* expression by inflammatory cytokines and its effect on *CSF2* expression in FLS**

(A) *F13B* expression levels in FLS stimulated with TNF or IL-6 plus IL-6Rα for 24 hours were detected by RT-qPCR (n = 3, each from an individual subject). Data were analyzed using Dunnett’s test. (B) FLS were transfected with F13B IVT mRNA or EGFP IVT mRNA for 24 hours. EGFP IVT mRNA was used as a negative control. *CSF2* expression levels in these cells were measured by RT-qPCR (n = 3). Data were analyzed using Student’s t-test. Abbreviations: TNF, tumor necrosis factor; IL-6, interleukin-6; FLS, fibroblast-like synoviocytes; CFH, complement factor H; EGFP, enhanced green fluorescent protein; F13B, factor XIII B subunit.
