# Supplementary material for: TNF-driven intracellular complement factor H–NF-κB feed-forward loop amplifies inflammatory mediator production in rheumatoid arthritis synovial fibroblasts
Source: Inflamm Regen. 2026 Aug 4;46:40. doi: 10.1186/s41232-026-00437-1 (PMC13721194; doi:10.1186/s41232-026-00437-1)
Supplement: Supplementary file 5 [file 41232_2026_437_MOESM3_ESM.docx]

**Additional File 3. Comparison of plasmid- and IVT mRNA-mediated the expression of FXIII-B and FH in MH7A cells**

(A–D) Immunocytochemistry of MH7A cells 24 hours after transfection. Cells were stained with an anti-HA tag antibody or an anti-EGFP antibody, followed by Alexa Fluor 647-conjugated secondary antibody and DAPI. Scale bar = 100 µm. (A) F13B-HA or EGFP plasmids. (B) CFH-HA or EGFP plasmids. (C) F13B-HA IVT mRNA or EGFP IVT mRNA. (D) CFH-HA IVT mRNA or EGFP IVT mRNA. (E, F, G) Western blotting analysis of MH7A cells transfected with IVT mRNA for 24 hours. (E) F13B-HA IVT mRNA or EGFP IVT mRNA. (F, G) Cells transfected with CFH-HA IVT mRNA or EGFP IVT mRNA. (F) Total cell lysate. (G) Cytoplasmic fraction. Abbreviations: HA, influenza hemagglutinin; DAPI, 4′,6-diamidino-2-phenylindole; GAPDH, glyceraldehyde-3-phosphate dehydrogenase.
