# Supplementary material for: TNF-driven intracellular complement factor H–NF-κB feed-forward loop amplifies inflammatory mediator production in rheumatoid arthritis synovial fibroblasts
Source: Inflamm Regen. 2026 Aug 4;46:40. doi: 10.1186/s41232-026-00437-1 (PMC13721194; doi:10.1186/s41232-026-00437-1)
Supplement: Supplementary file 6 [file 41232_2026_437_MOESM4_ESM.docx]

**Additional File 4. Plasmid-mediated expression of *F13B* or *CFH* fails to induce inflammatory gene expression**

RT-qPCR was performed to detect expression levels of various factors (e.g., *CSF2*, *CXCL8*, *TRAF1*, *CXCL1*, *CXCL2*, and *CCL3*) in MH7A cells that were transfected with *F13B* or *CFH* expression plasmids (n = 3). (A) *CSF2* (B) *CXCL8* (C) *TRAF1* (D) *CXCL1* (E) *CXCL2* (F) *CCL3*. In contrast to IVT mRNA-mediated expression, plasmid-based expression of F13B or CFH did not significantly affect inflammatory gene expression, indicating that intracellular retention is required for their functional effects. Abbreviations: CSF2, colony stimulating factor 2; CXCL8, C-X-C motif chemokine ligand 8; TRAF1, TNF receptor-associated factor 1; CXCL1, C-X-C motif chemokine ligand 1; CXCL2, C-X-C motif chemokine ligand 2; CCL3, C-C motif chemokine ligand 3.
