# Supplementary material for: TNF-driven intracellular complement factor H–NF-κB feed-forward loop amplifies inflammatory mediator production in rheumatoid arthritis synovial fibroblasts
Source: Inflamm Regen. 2026 Aug 4;46:40. doi: 10.1186/s41232-026-00437-1 (PMC13721194; doi:10.1186/s41232-026-00437-1)
Supplement: Supplementary file 7 [file 41232_2026_437_MOESM5_ESM.docx]

**Additional File 5. IVT mRNA-mediated expressions do not associate with alteration in endoplasmic reticulum stress markers**

(A, B) RT-qPCR was performed to assess the expression levels of endoplasmic reticulum (ER) stress-related factors in MH7A cells transfected with CFH IVT mRNA or EGFP IVT mRNA, or treated with tunicamycin for 24 hours (n = 3). (A) HSPA5 (B) DDIT3. (C) Western blotting analysis of eIF2α phosphorylation in MH7A cells transfected with CFH IVT mRNA or EGFP IVT mRNA, or treated with tunicamycin for 4 hours. Abbreviations: HSPA5, heat shock protein family A (Hsp70) member 5; DDIT3, DNA damage inducible transcript 3; eIF2α, eukaryotic translation initiation factor 2A.
