# Supplementary material for: TNF-driven intracellular complement factor H–NF-κB feed-forward loop amplifies inflammatory mediator production in rheumatoid arthritis synovial fibroblasts
Source: Inflamm Regen. 2026 Aug 4;46:40. doi: 10.1186/s41232-026-00437-1 (PMC13721194; doi:10.1186/s41232-026-00437-1)
Supplement: Supplementary file 8 [file 41232_2026_437_MOESM6_ESM.docx]

**Additional File 6. Gene ontology analysis of RNA-seq data shown in Figure 3A**

RNA was collected from FLS transfected with CFH IVT mRNA or EGFP IVT mRNA (negative control). RNA-seq analysis was performed to examine FH-induced gene expression in primary cultured FLS (n = 4, each from an individual subject). This table shows the list of functional annotations for upregulated genes. The ontology analysis was performed using DAVID Bioinformatics Resources v2025 (https://david.ncifcrf.gov/), accessed on July 7, 2026.
