# Supplementary material for: TNF-driven intracellular complement factor H–NF-κB feed-forward loop amplifies inflammatory mediator production in rheumatoid arthritis synovial fibroblasts
Source: Inflamm Regen. 2026 Aug 4;46:40. doi: 10.1186/s41232-026-00437-1 (PMC13721194; doi:10.1186/s41232-026-00437-1)
Supplement: Supplementary file 9 [file 41232_2026_437_MOESM7_ESM.docx]

**Additional File 7. NOD2 is detected in an FH-containing protein complex**

MH7A was transfected with CFH IVT mRNA. Cell lysates were immunoprecipitated with either an anti-HA-tag antibody or control IgG, followed by capture with Protein G beads. The eluted samples were analyzed using western blotting. Input and IP panels were exposed for different durations. The white arrowhead indicates the band corresponding to endogenous NOD2. Abbreviations: IP, immunoprecipitation; FH, factor H; NOD2, nucleotide‐binding oligomerization domain 2.
