# Supplementary material for: TNF-driven intracellular complement factor H–NF-κB feed-forward loop amplifies inflammatory mediator production in rheumatoid arthritis synovial fibroblasts
Source: Inflamm Regen. 2026 Aug 4;46:40. doi: 10.1186/s41232-026-00437-1 (PMC13721194; doi:10.1186/s41232-026-00437-1)
Supplement: Supplementary file 10 [file 41232_2026_437_MOESM8_ESM.docx]

**Additional File 8. FH-induced *TRAF1* expression is associated with increased MH7A cell proliferation**

(A, B) MH7A cells were transfected with CFH IVT mRNA, EGFP IVT mRNA, or mock IVT mRNA. (A) Cells were additionally treated with a NOD2 inhibitor (GSK717, a competitive antagonist). RT-qPCR showed that FH-induced TRAF1 expression was suppressed by GSK717 (n = 3, Dunnett’s test). (B) Cell viability assay using calcein-AM at 24 hours after transfection. Cell numbers in CFH IVT mRNA-transfected cells were higher than in controls (n = 6, Student’s t-test). (C, D) MH7A cells were transfected with 50 nM siRNA against CFH (siCFH) or control siRNA (siControl). Cells were harvested 96 hours after transfection. (C) RT-qPCR analysis showed decreased TRAF1 expression following CFH knockdown (n = 3, Student’s t-test). (D) Cell viability assay using calcein-AM. Cell numbers in siCFH-treated cells were lower than in siControl (n = 6, Student’s t-test).
