# Supplementary material for: TNF-driven intracellular complement factor H–NF-κB feed-forward loop amplifies inflammatory mediator production in rheumatoid arthritis synovial fibroblasts
Source: Inflamm Regen. 2026 Aug 4;46:40. doi: 10.1186/s41232-026-00437-1 (PMC13721194; doi:10.1186/s41232-026-00437-1)
Supplement: Supplementary file 11 [file 41232_2026_437_MOESM9_ESM.docx]

　　

**Additional File 9. Anti-FH antibody does not suppress TNF-induced cytokine gene expression**

(A–C) MH7A cells were transfected with either CFH IVT mRNA or EGFP IVT mRNA. Cells were also treated with anti-FH antibody, control IgG, and/or TNF. IVT mRNA was transfected for 10 hours, after which neutralization was performed, followed by the addition of TNF for 12 hours. The expression levels of the following cytokines were measured by RT-qPCR (n = 3, Student’s t-test): (A) *CSF2*, (B) *CXCL8*, and (C) *IL6*. Abbreviations: FH, factor H; IgG, immunoglobulin G.
